# A practical step-by-step approach for patient and public involvement in eHealth intervention research: Lessons learned from three case projects

**DOI:** 10.1016/j.invent.2025.100896

**Published:** 2025-12-03

**Authors:** Milon H.M. van Vliet, Roxy A. van Eersel, Charlotte C. Poot, Jasper S. Faber, Jiska J. Aardoom, Eline Meijer, Anke Versluis

**Affiliations:** a Department of Public Health and Primary Care, Leiden University Medical Center, Leiden, the Netherlands; b National eHealth Living Lab, Leiden University Medical Center, Leiden, the Netherlands; c Department of Human-Centered Design, Faculty of Industrial Design Engineering, Delft University of Technology, Delft, the Netherlands

**Keywords:** Patient and public involvement, Participatory research, End-user involvement, eHealth, Lessons learned, Recommendations

## Abstract

**Background:**

The importance of patient and public involvement (PPI) in research is increasingly acknowledged. PPI is a collaborative approach in which research is conducted with or by end-users. It can enhance research quality and benefit the involved end-users. However, involving end-users in the non-linear and often interdisciplinary process of eHealth development can be challenging. While many resources on PPI exist, a functional and practical overview tailored to eHealth research is lacking. This paper presents a step-by-step approach to support PPI implementation in eHealth intervention research.

**Methods:**

Three (ongoing) eHealth projects, each targeting a different population and applying different forms of PPI, informed the approach development. It was iteratively refined based on insights gained from these projects and feedback from other researchers and end-users involved in one of the projects.

**Results:**

A six-step approach was developed, each step accompanied by reflective questions to support preparation and evaluation. The steps are: (1) *Where in the eHealth evaluation cycle is your research project positioned?*; (2) *Why do you want to use PPI?; (*3) *Who is your target population?*; (4) *How are you going to achieve your aims?*; (5) *What considerations and conditions need to be taken into account to facilitate PPI?*; (6) *How did the PPI process unfold?* Each step includes recommendations, lessons learned, case examples, and relevant resources (e.g., literature, websites).

**Conclusion:**

The approach integrates literature with practical, field-based insights. We hope that the approach inspires and supports researchers in implementing meaningful PPI in research.

## Highlights

• We developed a practical approach to guide end-user involvement in eHealth research
• Six-step approach including reflective questions, recommendations and case examples
• Developed via an iterative process with input from researchers and end-users
• Supporting worksheet helps researchers apply the approach with relevant resources
• Practical tool to support and inspire meaningful patient and public involvement

## 1 Introduction

Patient and public involvement (PPI) is a collaborative approach in which research is carried out with or by end-users, such as patients, the public, and other relevant stakeholders (Health Research Authority, n.d.). PPI refers to consultation and collaboration in the design, conduct, and dissemination of research rather than participation as a study subject (Hughes and Duffy, 2018). Over the past two decades, the importance of PPI in research has become increasingly evident. By integrating the preferences and needs of people from the target population into the research design, PPI can improve the feasibility of procedures and enhance research quality (Harrison et al., 2019). In addition, PPI can benefit the relevance of research by aligning study aims and outcome measures with the real-life experiences of end-users (Brett et al., 2014a). It can also support the dissemination and implementation of results, both within and beyond the academic scope, by increasing credibility, validity, and sense of ownership (Baines et al., 2022; Brett et al., 2014a; Greenhalgh et al., 2019). Research involvement may also be directly beneficial for those engaged, for example, through skill-building and increasing self-worth (Brett et al., 2014b). Finally, literature often cites a moral aspect of PPI: the end-user has a right to give input on research that concerns them (Greenhalgh et al., 2019).

PPI is especially relevant in the development, evaluation, and implementation of eHealth interventions, as the end-user of the intervention is often the target population of the research. As such, PPI is valuable to address well-known challenges within eHealth research, such as high drop-out rates (Meyerowitz-Katz et al., 2020) and digital exclusion (van der Kleij et al., 2019). Many eHealth interventions have the potential to be tailored to individual users. PPI can further strengthen this by ensuring that interventions are aligned with the needs and preferences of end-users, thereby avoiding a one-size-fits-all approach (Al-Dhahir et al., 2022; van der Kleij et al., 2019). As a result, PPI can contribute to more inclusive eHealth interventions (Kilfoy et al., 2024). Such inclusivity is particularly important in the context of current health disparities, as interventions often do not reach or resonate with those who could benefit the most (Al-Dhahir et al., 2022; Marcolino et al., 2018). Additionally, the iterative process of developing and evaluating eHealth interventions, with multiple shorter development and evaluation cycles, provides many opportunities for PPI (Kip et al., 2025). To illustrate, end-users can provide input on different versions of the intervention, allowing researchers to integrate this input into the next cycle. Such continuous and dynamic PPI can improve the usability, engagement, and effectiveness of the intervention (Baines et al., 2022) and enhance successful implementation (Avila-Garcia et al., 2019).

Although PPI offers many potential benefits, it is often challenging to put it into practice, particularly in the context of eHealth research. Actively involving end-users in the non-linear process of the development and evaluation of eHealth interventions may be complicated and time-intensive (Brett et al., 2014a; van Schelven et al., 2020) and does not always match the more rigid and long-term characteristics of research. eHealth research also often requires an interdisciplinary approach, with stakeholders such as software developers whose priorities and needs may differ from those of researchers or end-users (Baines et al., 2022; Kip et al., 2025). Moreover, the implementation of PPI can vary widely depending on factors such as the target population, the specific phase of the eHealth evaluation cycle (Bonten et al., 2020) and the chosen form of involvement (Hughes and Duffy, 2018).

The use of PPI has increasingly been reported in major medical journals (Vanneste et al., 2025). Multiple frameworks for PPI in research have been published, such as the examples provided by Greenhalgh et al. (2019) and the person-based approach of Yardley et al. (2015). However, despite the specific challenges inherent to eHealth interventions, most existing frameworks were not originally conceived for use in eHealth settings. Moreover, the practical implementation is often described only in broad terms, lacking critical information on how and when to implement which methods (Kilfoy et al., 2024), limiting the opportunities for researchers to learn how to apply it meaningfully (Hughes and Duffy, 2018).

To address the gap between theory and practice, and to accommodate the characteristics and challenges of eHealth, we aim to develop a step-by-step, iterative approach offering practical, phase-specific guidance for applying PPI in specific research contexts. Each step is accompanied by reflective questions to help researchers prepare for and assess PPI, along with recommendations (i.e., practical suggestions for effectively carrying out each step) and lessons learned (i.e., successes and challenges) from three eHealth research projects. Additionally, we aim to identify and organize existing tools and resources relevant to each step, integrated into a self-developed worksheet. Ultimately, the goal of developing this approach is to provide a practical tool, raise awareness of potential challenges, make resources more accessible, promote knowledge sharing, and inspire researchers to implement PPI.

## 2 Methods

This paper follows the GRIPP2 short-form checklist (Staniszewska et al., 2017) to ensure comprehensive and transparent reporting of the PPI activities conducted to develop the step-by-step approach. The approach was developed by the author team – all eHealth intervention researchers who have prior PPI experience – and refined based on feedback from other researchers, as well as from end-users who had been long-term engaged in an eHealth intervention project. A detailed description of the PPI aim, methods, results, and reflections following the GRIPP2 short-form guidelines is provided in Appendix A.

### 2.1 eHealth intervention research case examples

Three (ongoing) eHealth projects have informed the development of the step-by-step approach: 1) the ‘Digital asthma medication adherence intervention’ project (Faber et al., 2023), 2) the ‘Data-supported treatment’ project (Versluis, 2025), and 3) the ‘Perfect Fit’ project (van Vliet et al., 2024; Versluis et al., 2024). Each project targets a different population and uses different forms of PPI. Table 1 provides an overview of the eHealth projects and a general description of the PPI activities conducted in each. The approach was iteratively refined based on experiences and insights gained throughout these projects. In addition, the projects serve to illustrate key experiences and lessons learned.

**Table 1:** Characteristics of the eHealth projects and general description of the conducted patient and public involvement (PPI) activities.

Case	Project aim	Target population	PPI
*Digital asthma medication adherence intervention*	Develop a digital medication adherence intervention for and with asthma patients with low health literacy.	Asthma patients with low health literacy.	The study employed a participatory design approach, structured around the five modes of design thinking: *empathize*, *define*, *ideate*, *prototype*, and *test*, applied across two iterative cycles. PPI was integrated into the *empathize* phase of the first iteration to gain a deeper understanding of user needs, and into the *test* phase of both iterations to assess the usability and acceptability of the prototypes.To ensure meaningful involvement of individuals with low health literacy (N = 5), the study applied participatory design methods specifically tailored to this group. These included co-constructing stories, experience prototype exhibitions, and video prototype evaluations—methods known to support engagement, comprehension, and feedback in this population.
*Data-supported treatment*	Examine the effectiveness and cost-effectiveness of an innovative, data-supported, personalized treatment for anxiety and mood disorders compared to usual care.	Adults with an anxiety or depressive disorder.	Three people (i.e., from here on referred to as patient representatives) affiliated with two national patient organizations contribute to the development and refinement of the research proposal. They share their input during group discussions within the consortium and in one-on-one meetings with the project leaders. They also provide written feedback. At the start of the project, the patient representatives supported the recruitment of a broader advisory group consisting of (ex)patients (n = 4–5). The patient representatives and the members of the advisory group are involved in all research stages of the project — from preparation to implementation– by providing input on study materials and procedures and participating in group discussions. At the start of each research phase, the patient representatives and members of the advisory group complete a participation matrix to outline individual contributions and responsibilities within the project. Their involvement is intended to increase the relevance and feasibility of the project and to help generate outcomes with practical value.
*Perfect Fit*	Develop, test, and evaluate Perfect Fit, an mHealth intervention with a virtual coach providing personalized support to promote smoking cessation and physical activity. We aimed to make Perfect Fit accessible and relevant for individuals with a lower socioeconomic position and/or eHealth literacy.	Adults (with lower socioeconomic position) who smoke daily and intend to quit within 6 weeks.	An advisory panel of potential end-users with experience or intent to quit smoking was established one year into the project. The panel consisted of three active members, with varying characteristics such as being 65+ years old, having little or no prior research experience, lower digital skills, or a lower socioeconomic position. The panel was involved throughout the project, providing input at various phases and for several substudies. Additionally, one-time PPI activities with other end-users were organized to gather further feedback on specific intervention components or research questions. For instance, focus groups were held with individuals aged 45 and older, with a low-to-middle socioeconomic position and insufficient physical activity levels, to discuss potential features of Perfect Fit. Experts (e.g., lifestyle coaches, psychologists), who may work with Perfect Fit in the future, were also consulted through interviews to incorporate their professional input into the intervention.

### 2.2 Developing the step-by-step approach

The step-by-step approach was developed by the author team using a reflective and iterative process, incorporating input from PPI activities involving (1) other public health and health psychology researchers and (2) end-users from the Perfect Fit project. The development was grounded in both practical experience and theoretical insights, drawn from the author team's evolving expertise and the input gathered through the PPI activities. Draft versions were reviewed and refined in multiple rounds to enhance clarity, completeness, and usability. This ultimately resulted in an approach with six steps, each accompanied by recommendations (i.e., practical suggestions for effectively carrying out each step), lessons learned (i.e., successes and challenges), illustrative case examples, and supporting resources.

#### 2.2.1 Input from the author team

The initial version of the approach, consisting only of the six steps, was drafted by CP and MV, based on their PPI experiences in different eHealth research projects. CP, RE, and MV also held multiple brainstorming sessions to identify initial recommendations and lessons learned. These were further expanded and refined by the full author team. Additionally, the team collected relevant resources (e.g., scientific literature, websites on PPI) and created illustrative case examples based on their own project experiences. Some of the authors received training or collaborated with Dutch organizations (e.g., INVOLV^2^, Longfonds^3^) to deepen their understanding of PPI and how to implement it in practice.

#### 2.2.2 PPI activities with other researchers

Intermediate versions of the approach were presented to other (eHealth) researchers during internal research meetings and at (inter)national Health Psychology and Public Health conferences. These sessions allowed the researchers to assess whether the approach was relevant, recognizable, and broadly applicable, and to identify any missing topics or additional insights.

#### 2.2.3 PPI activity with end-users Perfect Fit project

In the Perfect Fit case study, individual end-evaluation interviews were conducted with the three members of the end-user advisory panel. Open-ended questions were designed around key topics from the approach, such as collaboration experiences, freedom to express opinions, perceptions of contribution to the project, and the evaluation of recognition and rewards. The questions were further informed by the Dutch version of the Public and Patient Engagement Evaluation Tool (PPEET; Bavelaar et al., 2021). The input gathered from these interviews was used to verify whether all relevant topics were included in the step-by-step approach and to determine whether the feedback aligned with the recommendations and lessons learned, or whether revisions were required.

## 3 Results

### 3.1 Impact of PPI on the development of the step-by-step approach

The PPI activities, involving both other researchers and end-users from the Perfect Fit project, contributed to greater clarity, practical applicability, and better alignment with the needs and expectations of both researchers and eHealth end-users.

Feedback from researchers (via research meetings and conferences) confirmed the relevance and usability of the approach across contexts. It also led to specific additions, such as the inclusion of information on estimating necessary resources (e.g., budget) for meaningful PPI, in response to recurring questions. Furthermore, several researchers highlighted the challenge of evaluating PPI – an often-mentioned gap in the literature – which contributed to addressing this topic more explicitly in the approach. Ongoing reflection by the author team helped to embed researcher perspectives not only through external feedback, but also through internal reflection and iteration.

The individual interviews with the Perfect Fit end-users confirmed that the topics covered in the approach, recommendations, and lessons learned aligned well with their experiences. One new lesson was added based on these interviews: the importance of providing ongoing and explicit feedback to end-users on how their input has been used in the project.

Overall, the PPI process facilitated the iterative integration of perspectives from both public health and health psychology researchers and eHealth end-users, enhancing the robustness and relevance of the final approach.

### 3.2 Applying the step-by-step approach

We developed a practical six-step approach, accompanied by reflective questions for researchers. A worksheet was created to guide users through the steps and to provide relevant resources (e.g., scientific literature, PPI websites). Two versions are available: English (Appendix B), with only resources available in English, and Dutch (Appendix C), with additional Dutch resources.

#### 3.2.1 Recommended timing and use

The approach can be applied across multiple phases of an eHealth research project. We recommend starting using it in the early planning stages – during conceptualization, proposal development, and project setup. Reflecting on the steps and taking notes on PPI implementation can support the development of an initial PPI plan and help identify required resources (e.g., time, personnel, funding), facilitating their appropriate allocation. While early use is advised, the approach remains valuable even if adopted later. We recommend revisiting it throughout the project, as it may support concrete PPI planning, offer inspiration for shaping activities, and serve as a practical checklist. Documenting plans can also enable progress tracking and facilitate evaluation, both during and at the end of the project. Assessments of whether initially formulated goals, plans, and agreements have been achieved or require adjustments can be done individually or together with end-users.

#### 3.2.2 An iterative and reflective process

It is important to note that the approach is not static or linear; the steps are intended to be used iteratively. Designing and implementing PPI is an ongoing process that requires continuous reflection, evaluation, and adaptation. Depending on the project's timeline and PPI activities, it might be helpful to first outline a step in general terms and revisit it later. For instance, during intervention development, new questions may arise or intermediate evaluations may reveal that initial ideas have not yet been realized, prompting a return to earlier steps to refine or revise them. Compared to more standardized research methodologies guided predefined protocols, PPI is more dynamic, involves collaboration, and often follows a bottom-up approach. We therefore encourage researchers to maintain an open, flexible attitude and to trust the process as it unfolds.

#### 3.2.3 Adaptability across projects

We aimed to develop a broadly applicable approach that can be tailored to different researchers, contexts, and eHealth projects. However, some elements of the approach may be more relevant in certain contexts (e.g., depending on the research phase, or when using one-time versus ongoing PPI activities). Researchers are encouraged to adapt the approach to their situation and select the elements that best fit their needs.

### 3.3 The step-by-step approach

**Fig. 1:**
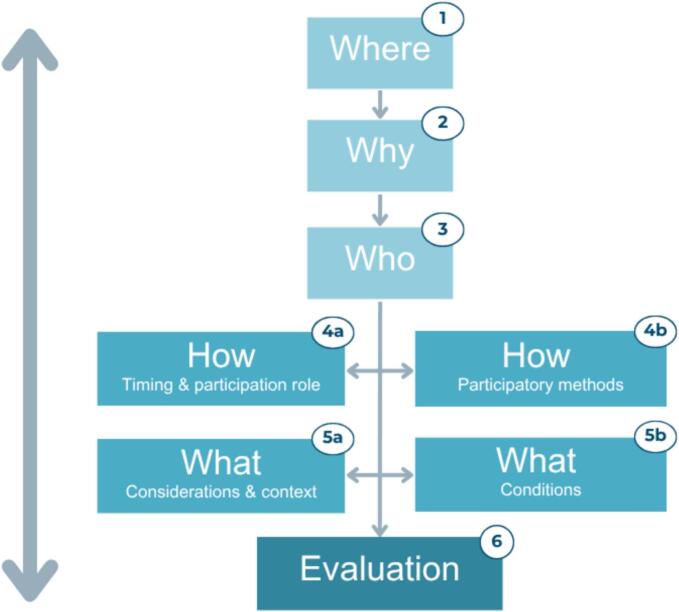
Overview of the six steps of the step-by-step PPI approach.

Fig. 1 presents an overview of the step-by-step approach, which is intended to guide researchers in preparing for and continuously reflecting on the implementation of PPI. The arrows in the figure indicate the iterative nature of the process. Below, each step is explained in more detail, accompanied by recommendations (i.e., practical suggestions for effectively carrying out each step) and lessons learned (i.e., successes and challenges), and illustrative case examples from three eHealth projects, which are outlined in Table 2, Table 3, Table 4, Table 5, Table 6. In addition, relevant resources to support each step are provided in the English and Dutch-language worksheets (Appendices B and C, respectively).

#### 3.3.1 Step 1: WHERE – Where in the eHealth evaluation cycle is your research project positioned?

This step invites researchers to consider the current research phase of their project. Identifying the phase helps define PPI goals, determine whom to involve, and decide how to involve them – topics explored further in the other steps of the approach. Table 2 presents recommendations, lessons learned, and illustrative case examples for step 1.

**Table 2:** ‘Step 1: WHERE - Where in the eHealth evaluation cycle is your research project positioned?’ – Recommendations, lessons learned, and case examples.

Recommendations and lessons learned	Practical case examples
**Identify the research phase to guide PPI planning** First, identify the current phase of your research project, for example, using the eHealth evaluation cycle (Bonten et al., 2020). Knowing the phase helps guide the subsequent steps of the approach.	*Case example: Digital asthma medication adherence intervention* The project started in the **conceptual and planning phase** of the eHealth evaluation cycle. Although the aim was to improve medication adherence among asthma patients with low health literacy, the researchers first needed to understand *why* nonadherence occurs in this group. Rather than moving directly into design, participatory methods were used to explore patients' beliefs, motivations, and barriers to clarify the problem before developing solutions. *Case example: Perfect Fit* Recruitment for the advisory panel took place during the **conceptual and planning phase**, with the intention of involving the panel throughout subsequent phases. When the panel was formed, the overarching goals, timeline, and general project scope had already been defined (i.e., the grant proposal had been approved). However, the design and content of the eHealth intervention, as well as the studies on its development, piloting, and evaluation, still needed to be determined. *Case example: Data-supported treatment* The eHealth platform used to deliver the data-supported treatment was already being utilized by multiple treatment centers before this research project. The current project, a randomized controlled trial, aims to evaluate the effectiveness and cost-effectiveness of data-supported treatment via this eHealth platform and is therefore in the **effectiveness (impact) phase** of the eHealth evaluation cycle.
**Involve end-users early** We recommend involving the target population as early as possible, as they are the best judges of their own needs and what is relevant to them. Early input can help shape intervention ideas, research projects, and research questions. However, even in later phases, involvement remains highly relevant and beneficial throughout the research cycle.	*Case example: Perfect Fit* In the Perfect Fit project, the decision to combine smoking cessation and physical activity in one intervention was made during the grant application stage, based primarily on prior research suggesting synergistic benefits of targeting these behaviors together. One end-user was involved in this early stage and later joined the advisory panel. However, the full panel was only established after funding had been secured, meaning most members were not involved in shaping the initial concept. Earlier involvement of the full panel could have enabled exploration of whether this behavioral combination matched their needs, or whether they would have preferred a different focus, such as smoking cessation alone or paired with another behavior. While such alternative suggestions were not explicitly raised during the project, the panel's later contributions demonstrated the potential value of earlier involvement.

#### 3.3.2 Step 2: WHY – Why do you want to use PPI?

This step encourages researchers to critically reflect on their reasons for involving end-users and define the aims of PPI within their project. PPI can serve multiple purposes and offers several benefits, including improving the feasibility and acceptability of study procedures, enhancing research quality (Harrison et al., 2019), and increasing the relevance and inclusivity of (eHealth) interventions (Al-Dhahir et al., 2022; van der Kleij et al., 2019) and the research process itself (Brett et al., 2014a). PPI can also benefit the involved end-users, for instance by increasing their skills and sense of self-worth (Brett et al., 2014b). Nowadays, many funders of research projects require involvement of the target population, which means that PPI can also be driven by extrinsic motivations. However, implementing PPI without a clear focus raises ethical concerns and should be avoided. Formulating clear aims not only helps to determine whom to involve and what activities or methods to use, but also facilitates ongoing monitoring of progress throughout the project. With well-defined aims, researchers can furthermore more easily monitor progress and assess whether adjustments are needed in aims, participants, planning, or methods. To guide this step, researchers can reflect on questions such as:

• *Why do you want to use PPI in your research project?*
• *What are the overall aims that you would like to achieve?*
• *What could be the added benefit of using PPI in your project?*
• *How do your PPI aims align with the broader objectives of your study?*

Table 3 provides further guidance for step 2.

#### 3.3.3 Step 3: WHO – Who is your target population?

This step prompts researchers to identify the end-users they want to involve, which is influenced by the characteristics of the research project's population and the PPI aims formulated in step 2. Key to selecting appropriate individuals is identifying those who can meaningfully contribute to achieving these aims and represent the project's target population. Different aims may call for involving different individuals, depending on the type of input or experience required. For instance, researchers may involve an advisory panel of end-users who are engaged throughout the project and become increasingly familiar with the research and its context. In parallel, other end-users may be involved in one-time PPI activities to provide fresh perspectives or because ongoing involvement would be too burdensome. To reflect on this step, researchers may consider the following questions:

• *Who is your target population?*
• *Who would benefit from your research?*
• *Who do you need to involve to achieve your PPI aims?*
• *Who can represent the population of your research project?*
• *What lived experiences or perspectives are essential to achieving your PPI aims?*
• *Are there groups that are often underrepresented but whose input is critical for your project?*

Since steps 2 and 3 are closely interconnected, Table 3 provides additional guidance for both these steps.

**Table 3:** ‘Step 2: WHY – Why do you want to use PPI?’ and ‘Step 3: WHO – Who is your target population?’ – Recommendations, lessons learned, and case examples.

Recommendations and lessons learned	Practical case examples
**Set realistic goals for PPI** Set realistic goals regarding what you want to achieve with PPI.	*Case example: Data-supported treatment* As the intervention was already developed before the study, PPI was not focused on designing or developing the intervention. The goals of PPI mainly focused on the research design (e.g., input on study procedures, co-creating study documents) with an emphasis on assessing the feasibility of procedures and the treatment approach, and ensuring the design and research materials better align with the needs and preferences of the study participants. Additionally, PPI supports communication and dissemination of the research findings.
**Ensure representative and inclusive PPI** Involve representatives of your research population. Try to involve a diverse and inclusive group of end-user representatives, which still matches the population they need to represent.	*Case example: Digital asthma medication adherence intervention* In this project, people with low health literacy were successfully involved through participatory methods chosen for their inclusivity and ability to facilitate expression of experiences, preferences, and needs. Visual probes—such as illustrated scenarios, story elements, and experience prototypes—enabled participants to share their experiences without relying solely on verbal communication. This approach helped reduce discomfort and encouraged more open, detailed responses. Additionally, immersive tools like experience and video prototypes allowed participants to physically or visually engage with potential technologies, making abstract concepts more tangible. These methods proved especially helpful for individuals with limited familiarity with digital health tools.
**Match end-users to your PPI aims** Match the end-users to your aims, considering: - The stages of the project: Involving different (types of) end-users at various stages of your project can be helpful and valuable for achieving multiple objectives. - The frequency and type of involvement: Decide if you need one-time or recurring involvement or both to achieve your aims. - The skills and interests of end-users: Select end-users based on the required skills and scope of your aims.	*Case example: Data-supported treatment* Within the project, we involved patient organization representatives and end-users in an advisory group. The patient representatives, who had experience with scientific research from a PPI perspective, were more closely involved with the trial and collaborated with researchers on more technical aspects. They contributed a collective perspective, whereas the end-users provided input based on their individual perspectives and experiences.
**Collaborate with patient organizations** If the research project focuses on a patient population, consider involving representatives of relevant patient organizations. Consult networks to find organizations representing your research population.	*Case example: Data-supported treatment* Patient representatives have been involved as project partners from the start, including writing the funding proposal. They have recruited end-users from within the organizations for involvement in the advisory group for this project. As the patient representatives had prior experience with scientific research, they were well-suited to assist with implementing PPI practices, facilitating the collaboration between researchers and end-users.

#### 3.3.4 Step 4: HOW – How are you going to achieve your aims?

This step supports researchers in planning and organizing PPI activities to achieve their aims. It consists of two components: (a) *Timing (research phase) and participation role,* and (b) *Participatory methods.*

The first component (a), partly inspired by the ‘Involvement Matrix’ tool (Kenniscentrum Revalidatiegeneeskunde Utrecht, 2019; Smits et al., 2020), helps researchers determine *when* to involve end-users and *in what role*. Researchers are encouraged to create a preliminary overview of participatory activities (e.g., inviting end-users to advise on recruitment strategies or co-create intervention design) across the different stages of the research project – preparation, execution, and completion (as defined in the Involvement Matrix). For each activity, it is helpful to consider the role of involvement that end-users will take on. The Involvement Matrix was specifically developed to support this process. Researchers are strongly encouraged to complete or discuss this overview collaboratively with end-users – either individually or as a group – to gain insight into, and incorporate, their ideas, needs, and expectations (Kenniscentrum Revalidatiegeneeskunde Utrecht, 2019; Smits et al., 2020). Reflective questions to guide this component include:

• *When will you involve your end-users?*
• *Which decisions or stages in your project would benefit from end-user input?*
• *Do you need continuity in involvement (*e.g.*, an advisory panel) or one-time involvement?*
• *In what ways can you involve them?*
• *What involvement role is desirable and achievable for end-users in each activity?*

After creating an overview of participatory activities, the second component of this step (b) prompts researchers to consider appropriate participatory tools and methods to achieve their PPI aims. Common research methods, such as interviews or focus groups, may be suitable for capturing end-users' perspectives. However, their effectiveness depends on how well individuals can articulate their needs, preferences, and experiences. To better capture the voices of end-users – especially when verbal expression is limited – interactive and creative approaches, such as storyboarding or prototype development, can be valuable. Tools should be tailored to the characteristics of the end-users, including their cognitive and communication abilities, cultural background, and lived experiences. This enhances inclusivity and improves the quality of involvement. Moreover, interactive methods can make participation more enjoyable and foster stronger collaboration and engagement among end-users. Immersing themselves in the end-users' world can further help researchers understand daily realities and align the research more closely with the population's needs. Finally, researchers should reflect on whether end-users are expected to share personal experiences or those of the broader population they represent. To support reflection, researchers may consider the following questions:

• *How will you recruit the people you need?*
• *How will you capture their voice?*
• *Will they speak from personal experience or as representatives?*
• *Which participatory tools and methods are most appropriate?*
• *How accessible are these methods to the end-users you intend to involve?*

Table 4 provides further guidance for both components of step 4.

**Table 4:** ‘Step 4: HOW – How are you going to achieve your aims?’ – Recommendations, lessons learned, and case examples.

(a) Timing (research phase) and participation role	
Recommendations and lessons learned	Practical case examples
**Plan participatory activities per research phase** Consider which research activities and processes need to take place in the current research phase, for instance, by using the involvement matrix (Kenniscentrum Revalidatiegeneeskunde Utrecht, 2019). Distinguish between activities for the preparation, execution, and completion of your research project(s). Then, create a preliminary overview of the research activities in which you can and want to involve your end-users through participatory activities. Examples of participatory activity formats include individual or group meetings, creative sessions, email exchanges, and questionnaires.	*Case example: Data-supported treatment* An overview of research activities per research phase was desired to identify possibilities for collaboration, as most end-users were unfamiliar with conducting research. By presenting the research activities per phase, the end-users could provide input on which activities they were interested in. Moreover, the overview enabled end-users to identify possibilities for collaboration that were previously overlooked by the researchers.
**Define roles per activity together with end-users** Make an overview of the role of each end-user in every participatory activity. The involvement matrix is a useful tool for this. Defining these roles in collaboration with the end-users allows for alignment with their ideas, needs, and expectations. If there are more suitable volunteers for certain research activities than required, activities or tasks can be collaboratively assigned based on individuals' strengths and interests.	*Case example: Data-supported treatment* Each end-user used the involvement matrix in collaboration with the researcher to define their role in each stage of the research. For example, at the start of involvement, end-users indicated to what extent they wanted to be involved in writing the information letters or designing the study procedures. At the start of the execution phase, they reevaluated their intended involvement in the upcoming participatory activities.


(b) Participatory methods	
Recommendations and lessons learned	Practical case examples
**Align expectations through introductory meetings** Since end-users do not always have prior experience with research involvement, they often do not know what to expect. Therefore, it can be helpful to first schedule individual introductory meetings with end-users to get to know each other, provide a brief overview of the research project, and gain insight into their experiences, expectations, and expertise. Then, in a group meeting, you can further align expectations and roles based on end-users' preferences, skills, and expertise, benefiting from the prior insights into their background to tailor the discussion accordingly.	*Case example: Perfect Fit* At the start of the collaboration with the advisory panel, a group meeting was organized to get to know each other (through an icebreaker activity), provide a brief introduction to the Perfect Fit project, and discuss key aspects of the collaboration. Topics addressed included expectations, desired levels of involvement (based on the Involvement Matrix roles; Kenniscentrum Revalidatiegeneeskunde Utrecht, 2019), what end-users hoped to gain from the collaboration, available time commitments, and preferred communication methods. These discussions were essential to align the collaboration with factors such as end-users' availability, physical mobility, and digital skills, while ensuring it fit within the overall research timeline, resources, and tasks. Later in the project, when a new advisory panel member joined, an individual introductory meeting was conducted. This one-on-one format allowed for a more extensive introduction, giving the end-user more space to share their background and ask questions. Based on this experience, it is recommended to schedule individual introduction meetings first, followed by a joint meeting to align expectations, roles, and practical matters.
**Clarify whose voice is represented** Think about whether you are capturing the personal voice of the end-user or of the people they represent. Which voice (personal or representative) is desired may depend on the research activity or the aim of PPI. An end-user could use tools to become more familiar with the perspectives of the people they represent, e.g., they could use surveys to gather input from the relevant patient community.	*Case example: Data-supported treatment* In the preparation phase of the trial, there was a discussion on using a secured email service. Two patient representatives stated that *personally* they would not mind receiving emails without this security measure, but they both believed the broader community of end-users would generally prefer the addition of the security measure. The patient representatives and researchers were aware of which voice they were capturing, which facilitated decision-making.
**Tailor meeting formats to end-user needs** Arrange accessible and regular meetings by tailoring modalities (e.g., in-person meetings, video calls, email exchanges) to the nature of the collaboration, and the availability and needs of end-users. Consider factors such as health literacy, mental health challenges, digital access, physical mobility, competing commitments (e.g., work, caregiving), and prior experiences with health care or research. In some cases, building mutual trust may be necessary before meaningful collaboration can take place. Proactive strategies – such as meeting in familiar settings or using personal introductions – can help create a safe and inclusive environment.	*Case example: Digital asthma medication adherence intervention* Prototype testing of a digital asthma adherence intervention was conducted in a separate room of the participating General Practice. Individuals with low health literacy were invited by the practice nurse, a trusted figure within the community, who also explained the purpose of the testing. Trust-building strategies were further integrated during the final prototype phase. Prior to the assessment, participants received a video message from the researcher, expressing appreciation for their involvement and outlining what to expect during the evaluation of the prototype's readability, usability, and navigability from the end-user perspective. *Case example: Data-supported treatment* As members of the advisory group were located throughout the country and had some reservations towards travelling relating to the nature of the target population (i.e., anxiety symptoms); there was a strong preference for online meetings through video calls.
**Use creative methods to surface implicit needs** Employ participatory design methods to uncover implicit desires and needs (i.e., what people know, feel, and dream), rather than focusing solely on explicit behaviors, actions, and verbal expressions (i.e., what they think, do, and say). This is especially useful when cultural or language gaps exist, as participatory design's hands-on, collaborative approach can reveal deeper, more authentic insights.	*Case example: Digital asthma medication adherence intervention* Participatory design methods were used to uncover the underlying, unarticulated needs and motivations of asthma patients with low health literacy. The researchers employed specific participatory methods that allowed patients to engage with abstract concepts in concrete ways. Co-constructing stories, for example, involved visual storyboards of fictional characters to prompt discussion and reflection on own behavior and motivations in using their asthma inhaler. A prototype exhibition, which included mock-ups of possible concepts, such as an augmented reality T-shirt, further supported participants in expressing their attitudes and preferences towards possible technologies and designs.
**Step into the world of your end-users** In addition to research-focused participatory activities, researchers are encouraged to empathize with end-users by immersing themselves in their world (i.e., the target population). Do not bring the end-user into your research world; instead, as a researcher, spend time in the end-user's world. This does not always require a structured activity; sometimes, simply spending a few hours with the end-user (e.g., accompanying them to a hospital appointment or participating in a daily activity like grocery shopping, depending on your research interest) can provide valuable observations for the research question and help better align with the end-user's lived experience.	*Case example: Perfect Fit* To better understand the needs and preferences of people with a lower SEP who smoke and increase the accessibility of the eHealth intervention for individuals who are often not reached by existing interventions, a day was spent at a community center regularly attended by one of the advisory panel members. During this visit, the activities of the advisory panel member were observed, and conversations were held with people who smoke from various (socio-economic) backgrounds. These interactions provided valuable insights into the lived experiences of the target population. For example, it became clear that the health risks of smoking — often emphasized in research and literature as important motivators for quitting — were not the primary concerns of those spoken to. Instead, issues like financial stress and family problems were often at the forefront, and smoking was seen as a way to alleviate stress. This experience highlighted the need to address social environmental factors and stress-inducing issues in our eHealth intervention.

#### 3.3.5 Step 5: WHAT – What considerations and conditions need to be taken into account to facilitate PPI?

This step prompts researchers to reflect on the characteristics of their research project that need to be considered when implementing PPI, as well as the conditions required to enable meaningful collaboration. It consists of two components: (a) *Considerations and context of the research project*, and (b) *Conditions for successful collaboration.*

The first component (a) encourages researchers to consider how specific characteristics of their research project might influence the planning and implementation of PPI. It can be helpful to reflect on aspects such as the research population, the composition and size of the research team, other stakeholders who need to be involved or informed (e.g., when developing an intervention together with software developers), and the timeline and budget. For instance, a large research team across multiple institutions requires a clear role distribution (e.g., who organizes PPI activities, who serves as the contact person for end-users). It is also important to establish communication and feedback loops to ensure that all team members are informed of insights gained through PPI and can identify opportunities where end-user involvement is valuable. To guide this component, researchers can reflect on questions such as:

• *What characteristics of the research project should be considered when planning and implementing PPI?*
• *What is your timeline and available budget?*
• *How is your research team composed, and who will be responsible for organizing and facilitating PPI?*
• *How will internal communication and feedback loops be established to share and act on PPI insights?*
• *Are there other stakeholders whose input or approval is necessary (*e.g.*, software developers)?*
• *Are there important collaborations with other parties that may affect PPI?*

The second component (b) focuses on preconditions, arrangements, and agreements that support meaningful and equitable collaboration with end-users. Harrison et al. (2019) provide a useful overview of principles and best practices to promote fruitful PPI collaboration. For example, researchers should recognize end-users as experts and ensure they feel valued and empowered. This may involve offering training or support to help them develop the knowledge and skills needed to engage in PPI activities. Additionally, researchers are encouraged to establish clear expectations, roles, and limitations, together with the involved end-users. Such arrangements contribute to building trust and promoting collaboration based on respect and equity. It is important to continuously evaluate the collaboration process and make adjustments as needed to maintain meaningful and effective involvement. Reflective questions to guide this component include:

• *What is expected from each stakeholder involved in PPI?*
• *What are the roles, rights, and responsibilities of researchers and end-users?*
• *What conditions are necessary to enable a fruitful collaboration?*
• *What practical arrangements are required (like logistics, budget, and compensation)?*
• *What kind of support or resources do end-users need to participate meaningfully?*
• *What strategies can you use to build trust and maintain engagement over time?*

Table 5 provides additional guidance for both components of step 5.

**Table 5:** ‘Step 5: WHAT – What considerations and conditions need to be taken into account to facilitate PPI?’ – Recommendations, lessons learned, and case examples.

(a) Considerations and context of the research project	
Recommendations and lessons learned	Practical case examples
**Adapt participatory activities to context and goals** Adjust the participatory activities to: - the target population of the research project as represented by the involved end-users (e.g., visual methods, like video prototype evaluation, for end-users with lower literacy). - the research phase. - the aims you want to achieve.	*Case example: Digital asthma medication adherence intervention* The study was carefully designed to be accessible for participants with low health literacy by simplifying content and using visual, intuitive formats. Instead of relying on written materials, the researchers used visual storyboards to present fictional characters and relatable scenarios, making it easier for participants to engage without the need for abstract or verbal reasoning. To introduce the study, the researcher replaced the traditional participant information letter with a short video in which he explained the research purpose and process in simple, conversational language. Later, during the video prototype evaluation, an animated video was used to clearly demonstrate the intervention concept, allowing participants to understand the functionality and purpose without requiring complex reading or technical explanations. These adaptations helped reduce cognitive barriers and created a more inclusive and comfortable environment for meaningful participation.
**Coordinate communication and feedback** Create communication flows and feedback loops. It is advisable to organize participatory activities by one or two “PPI coordinator(s)” to ensure continuity in the collaboration and facilitate relationship building between the coordinator(s) and the end-users. However, research projects often involve a research team and multiple stakeholders (besides end-users). Ensure that the coordinators know what to discuss with end-users on behalf of the entire research team and effectively communicate the feedback of end-users to the research team.	*Case example: Perfect Fit* At the start of setting up the advisory panel collaboration, two researchers who had a central role in the content development of the eHealth intervention were appointed as PPI coordinators. These coordinators organized the advisory panel collaboration and facilitated communication between the research team and the advisory panel. They regularly checked with the research team to identify agenda items or questions for the advisory panel, shared minutes and key input from advisory panel meetings with the researchers, and communicated important developments back to the advisory panel. They also ensured that proposed ideas were feasible within the limits of available resources and research expertise. In some meetings, the PPI coordinators invited other research team members to present or participate when the topic aligned with their work, such as a demonstration of the first prototype by a software developer.
**Plan resources and budget for meaningful PPI** Ensure adequate budget and time for engagement activities. Take into account compensation for end-user involvement and training, as well as the financial and time-related costs of preparing and executing the planned engagement activities. It helps to plan the involvement of the target population early in the project – preferably during the grant application stage – so that you can account for it in the planning and budget. This also ensures that the perspective of the target population is taken into account in an early stage (e.g., when formulating research questions) and that the involved individuals get to know the project well. If applicable, it is advisable to involve patient organizations in setting up the timeline and budget. They can offer input based on experience with PPI in previous research projects and often provide standard rates or guidelines for compensation.	*Case example: Digital asthma medication adherence intervention* Involving people who are considered disadvantaged requires time. The researchers initially focused on building trust by being present in community settings, engaging in informal conversations unrelated to the research topic, and participating in local activities. This approach led to the involvement of two individuals with both low health literacy and asthma. Trust-building was essential to ensure participants felt respected, heard, and safe to share personal experiences and stories. Due to time constraints, it was not possible to fully extend this process. To help bridge the gap between researchers and participants, research nurses played a key role as trusted intermediaries. *Case example: Perfect Fit* In the Perfect Fit project, there was sufficient budget, but, in hindsight, not enough time allocated for PPI. This was partly due to the uncertainty around how to implement PPI in a meaningful way, which led to delays in other research tasks. Planning PPI as early as possible can help prevent this. *Case example: Data-supported treatment* The funder of this research project required involvement of the target population, so PPI plans had to be included in the research proposal. Patient representatives provided input on this inclusion to ensure that the plans were not solely designed to secure funding but were genuinely aimed at implementing meaningful PPI throughout the project. As a result, the plans were defined early, enabling the timely allocation of budget and resources, and increasing their overall feasibility.


(b) Conditions for successful collaboration	
Recommendations and lessons learned	Practical case examples
**Discuss roles, expectations, and limitations** Make clear agreements upfront on the roles and responsibilities of all stakeholders involved and align expectations (e.g., make clear whether the aim is to represent the target population or to share personal perspectives). Establish ground rules collaboratively to allow shared authority and flexibility, while respecting that involvement is often voluntary and should accommodate end-users' preferences and capacities. Be transparent about potential limitations (e.g., budget, software development, or regulatory constraints) that may prevent full incorporation of all input. At the same time, researchers are encouraged to remain open and responsive to feedback, willing to adapt plans when appropriate. Recognize that PPI is an iterative, collaborative process in which researchers do not need all answers from the start but should co-create the process with stakeholders through ongoing preparation and collaboration.	*Case example: Data-supported treatment* During the preparation phase of the project, end-users provided input on the patient information letter, such as removing redundancies and rephrasing text to improve readability. However, upon submission to the ethics review board (METC), we were instructed to adhere to the required template, meaning some of their suggestions had to be reversed. We communicated these constraints back to the end-users. In retrospect, it might have been beneficial to explicitly address, from the outset, that certain guidelines or regulations can override input from end-users. This could help manage expectations and clarify the boundaries of PPI in regulated aspects of research. *Case example: Perfect Fit* During an introductory meeting with the advisory panel members, key aspects of the collaboration were discussed, such as expectations, desired roles and levels of involvement (based on the Involvement Matrix roles; Kenniscentrum Revalidatiegeneeskunde Utrecht, 2019), available time, and preferred communication methods. Discussing these topics together helped not only to align expectations but also to co-create the collaboration and establish ground rules. This ensures that the collaboration fits the needs of all involved stakeholders and allows everyone to feel a sense of agency and responsibility in the collaboration. After the introductory meeting, the researchers summarized the agreements in a collaboration agreement, which end-users reviewed, made adjustments to as needed, and signed.
**Ensure openness, trust, and adaptive collaboration** Ensure equitable power, trust, transparency, respect, and openness between end-users and researchers through bidirectional and open communication, shared decision-making, and valuing each other's input. Use lay language, avoid jargon, create a comfortable atmosphere, listen actively, and be transparent about what is done with feedback from the stakeholders. Regular face-to-face meetings, small groups, and informal social events can help ensure everyone has a chance to speak. This not only helps build relationships but also strengthens collaboration and facilitates communication.	*Case example: Digital asthma medication adherence intervention* Throughout the project, responsible and meaningful user involvement was a central principle. In the initial phase of the study, aimed at gaining a deeper understanding of the target population, participants were actively engaged within their own everyday contexts. For example, researchers spent time joining participants during their work at a local thrift store. This approach contributed to building trust and narrowing the gap between researchers and participants. Moreover, such a bottom-up engagement strategy proved valuable for establishing connections and fostering trust with other potential participants within the community.
**Enable informed and confident participation** Provide training and support for end-users and researchers. Ensure that end-users get to know the research project, know what PPI entails, and feel capable of sharing their opinion. Also, ensure that the research team becomes familiar (e.g., through training) with PPI. This will help researchers to achieve fruitful collaboration and enhances the participation and engagement of end-users.	*Case example: Perfect Fit* At the start of the collaboration and throughout the advisory panel's involvement, panel members were consistently kept well-informed. This included providing project updates, sharing necessary background information in clear, accessible language before participation activities, clearly explaining the expectations for advisory panel members, using accessible participatory activities, and offering ample opportunities for questions. The advisory panel appreciated this approach. Additionally, the panel was invited to annual research consortium meetings, occasionally received media releases or scientific posters about the project, and was encouraged to follow the project's social media pages. This allowed the advisory panel to stay updated on the project without requiring additional time from the researchers.
**Give concrete and timely feedback on input** Provide (ongoing) feedback to end-users on how their input has been utilized. After participatory activities, share feedback (e.g., via email or in the next meeting) on what has been done with their suggestions, so they can see the concrete impact of their contributions and feel heard and valued. It is also important to clarify in advance that not all suggestions may be implemented. When providing feedback, explain why certain suggestions were not feasible.	*Case example: Perfect Fit* Throughout the advisory panel collaboration, the researchers dedicated some time during each meeting to provide updates on the project, such as achieved milestones within the research projects or intervention development. This aimed to keep the panel informed about the project and highlight the progress made with the help of the advisory panel. However, the final evaluation revealed that while advisory panel members felt heard and valued, and believed they had contributed, they found it difficult to pinpoint or articulate their specific, concrete contributions to the project when reflecting on the collaboration. Based on this experience, it is recommended to provide short-term feedback, clearly outlining what has been done with their input, and explaining why some suggestions were not implemented (e.g., through minutes sent to end-users via email).
**Value, acknowledge, and compensate end-users** Think about compensation for end-users. Acknowledge them for their time and efforts, by expressing appreciation (e.g., compliments), providing rewards (payment, gift cards or gifts), acknowledging their contributions in end-products or output (e.g., publications, presentations, etc.), and ensuring that participating is also informative and fun (e.g., organizing informal and fun meetings). This will promote ownership and empowerment.	*Case example: Perfect Fit* Throughout the advisory panel collaboration, the researchers made efforts to regularly acknowledge the advisory panel members' contributions. For instance, they expressed appreciation for their valuable input, emphasized that their feedback contributed to the project, and highlighted their contributions during (scientific) presentations and in articles. As an example, during a Perfect Fit consortium meeting, the researchers gave a presentation about PPI within the project and the valuable collaboration with the advisory panel. One of the advisory panel members co-presented, sharing their experiences and perspectives firsthand. Additionally, to compensate for their time, financial compensation and small gifts were provided. The researchers also ensured that end-users were well-informed from the outset about what they could expect in return for their involvement, helping to set clear expectations. The advisory panel members also indicated that they found value in the collaboration, citing the new knowledge and skills they gained, the social connections it fostered, and the sense of contributing to something meaningful.
**Support sustainable collaboration** Think about how to maintain continuity of the collaboration, beyond ensuring a fruitful collaboration. Especially in longer-term projects or when working with more vulnerable end-users, stakeholders (including end-users and researchers) may stop involvement for any reason (e.g., health reasons, time restraints, job change, relocation). Anticipate how to handle such changes in involvement. Clear documentation of agreements and what information and supporting materials have already been shared with end-users may help maintain continuity in engagement. When recruiting new end-users to be involved, you can have an individual intake with them to bring them up to speed and allow them to get familiar with the project. Subsequently, they can be integrated into the ongoing group of involved end-users.	*Case example: Data-supported treatment* To maintain end-user engagement, we tailored the involvement process to the needs and preferences of the end-users as much as possible. This included the planning and format of meetings, how updates were shared, and communication methods throughout the project. Additionally, meetings began with informal check-ins. All involved people would occasionally receive a small gesture, such as chocolate and a card with a thoughtful message, both as a token of appreciation and to help keep the project on their radar and maintain motivation. For reasons unrelated to the collaboration, there were changes in involvement within different groups of stakeholders: the research team, the representatives from patient organizations, and the end-users. Due to having people leave and others taking over, there was some delay and inefficiency in involvement activities.

#### 3.3.6 Step 6: EVALUATION – How did the PPI process unfold?

This final step invites researchers to monitor and evaluate the PPI process and its impact, both during and after the collaboration. Ongoing reflection with end-users helps identify areas for improvement, acknowledge progress, and contribute to stakeholders feeling heard and involved. Additionally, researchers are also encouraged to conduct an end-of-project evaluation, ideally by revisiting the original PPI aims and agreements made earlier in the process. For instance, if agreements were made about providing training or giving feedback on how input was used, the evaluation can explore whether these agreements were met according to both end-users and researchers. Existing evaluation frameworks or tools, such as the Public Involvement Impact Assessment Framework (PiiAF; M. Collins et al., 2018; Popay et al., 2014) or the PPEET (Abelson et al., 2016; Bavelaar et al., 2021), can support this process. It is important to acknowledge that the specific impact of PPI is often difficult to measure or isolate. Research on how to evaluate its (added) value is ongoing (Boivin et al., 2018). Rather than focusing solely on quantifiable outcomes, we encourage researchers to view PPI as an iterative and relational process, in which value may lie more in the quality of engagement, mutual learning, and responsiveness throughout the project. Furthermore, to promote long-term impact, researchers can consider how to sustain the knowledge, methods, and networks developed through PPI beyond the project. This may include sharing resources openly, connecting interested end-users to future research, or embedding PPI more structurally through approaches such as Community-Based Participatory Research (S.E. Collins et al., 2018). To guide this step, researchers can reflect on questions such as:

• *What aspects are important to evaluate for both you and the end-users?*
• *How is the PPI process progressing?*
• *Are the predefined PPI aims being met?*
• *Does the process align with everyone's expectations and needs?*
• *Are there areas for improvement or elements that should be maintained?*
• *What has been the perceived added value of PPI so far?*
• *How can the outcomes or relationships from this collaboration be sustained?*

Table 6 provides further guidance for step 6.

**Table 6:** ‘Step 6: EVALUATION – How did the PPI process unfold?’ – Recommendations, lessons learned, and case examples.

Recommendations and lessons learned	Practical case examples
**Integrate ongoing monitoring and evaluation** Monitoring and evaluating the PPI process throughout the project – not just at the end – is crucial. Ongoing evaluation helps identify areas for improvement in a timely manner and ensures that all involved parties feel heard. It can also enhance the sense of shared decision-making and ownership over the process and ultimately, the quality of the research project.	*Case example: Perfect Fit* At the start of each meeting with the Perfect Fit advisory panel, a short reflective moment was included to check in on how everyone was doing and how the collaboration was progressing from everyone's perspectives. This allowed advisory panel members to share feedback on what was going well and what could be improved. Looking back, since the project spanned several years, it might have been helpful to also organize a more dedicated evaluation session halfway through. This could have allowed for a deeper reflection on the PPI aims, outcomes, roles, expectations, and collaboration.
**Conduct an end-of-project evaluation** Evaluation at the end of the collaboration is recommended to evaluate the process and perceived impact. Start by considering the purpose of your evaluation: what do you hope to learn, and what outcomes would be meaningful? Then decide which methods fit this aim (e.g., surveys or structured feedback sessions) and consider whether individual or group-based evaluation is most appropriate, as each may yield different insights.	*Case example: Perfect Fit* At the end of the project, individual evaluation sessions were held with each member of the advisory panel. The sessions had two goals: to discuss the preliminary findings of the evaluation study of the eHealth intervention and to reflect on the collaboration throughout the project. The researchers conducted individual semi-structured interviews, using open-ended questions based on the current approach, the Public and Patient Engagement Evaluation Tool (PPEET; Abelson et al., 2016; Bavelaar et al., 2021), and the agreements made in the collaboration agreement that was collaboratively developed at the start of the project. This approach allowed for in-depth reflection on the process and collaboration, helping to identify both strengths and areas for improvement.
**Carry forward knowledge and collaboration** Consider ways to sustain and share the knowledge, methods, and networks developed through PPI collaboration. This can include making working documents and methodologies openly accessible (e.g., Open Science), reporting approaches in (non-)scientific output, and presenting insights to colleagues. Encouraging institutional adoption by motivating colleagues to integrate PPI practices more broadly within the department can also support sustainability. Additionally, if end-users express interest in continued involvement, facilitating their engagement in other research projects seeking end-user input can help maintain engagement. For researchers aiming to embed PPI more structurally, approaches such as Participatory Action Research or Community-Based Participatory Research can serve as valuable inspiration.	*Case example: Digital asthma medication adherence intervention* To support knowledge dissemination among stakeholders directly involved in the study, a concise and visually accessible one-page summary was developed at the conclusion of the research. This document synthesized the main findings and the resulting intervention in a format tailored for practical use. It was shared with the health care professional who had played a key role in facilitating participant recruitment for the evaluation study. Rather than relying solely on the academic publication—which is typically text-dense, lengthy, and often not available until well after project completion—this summary was intended to provide a timely, user-friendly alternative for communicating outcomes to practice-based collaborators. *Case example: Current paper* This paper itself serves as a means to share and sustain knowledge gained through PPI in eHealth research projects. In response to the fragmented PPI resources and the challenge of knowing when and how to use them effectively in different project contexts, the author team developed the current approach. By sharing this approach through this scientific article and presenting it at conferences, the authors aim to support and inspire other researchers to plan, implement, and evaluate meaningful PPI, and to promote continued knowledge exchange and capacity-building within the research community.

## 4 Discussion

The importance of PPI is increasingly recognized. Yet its practical implementation remains challenging, partly due to a lack of structured overviews of information and resources to translate PPI principles into action. To help bridge this gap between theory and practice, we developed a step-by-step, iterative approach for PPI in eHealth intervention research. The approach provides practical, phase-specific guidance on planning and applying PPI in diverse research contexts. Each step is accompanied by reflective questions, as well as recommendations (i.e., practical suggestions for effectively carrying out each step) and lessons learned (i.e., successes and challenges) from three eHealth research projects. In addition, we provided an overview of existing resources to support each step. These steps, reflective questions, and resources were integrated into a practical worksheet for researchers to apply PPI to their own projects.

We encourage researchers to apply the approach as early as possible in the research process. PPI is a collaborative journey in which researchers are not expected to have all the answers from the start. Instead, the process is co-created with stakeholders. The steps offer structure, yet are intentionally flexible: the process is rarely linear in practice, and there is no one-size-fits-all model. It is also possible to have multiple PPI efforts going on simultaneously. To illustrate, there could be end-users involved throughout the entire project, while others are involved on an incidental basis. In addition to end-users of an eHealth intervention, projects may involve a range of other stakeholders, such as health care providers and health care managers, whose involvement may vary depending on the project's specific characteristics. Therefore, we encourage researchers to use the approach iteratively and tailor it to the specific context and needs of their project.

Our approach stands out for its strong foundation in both theory and practice. It was developed using a reflective and iterative process that drew on the evolving expertise of the author team and was further informed by insights from three eHealth research projects. Input from PPI activities involving other public health and health psychology researchers, as well as end-users from one of the case examples (i.e., the Perfect Fit project), helped align the approach with the needs and preferences of both researchers and end-users. These activities included presenting intermediate versions of the approach to (eHealth) researchers and interviewing end-users, which further enhanced its relevance, broad applicability, and completeness. By integrating challenges and insights from real-world projects, the approach captures valuable field-based knowledge that remains scarce in formal eHealth literature.

It is important to note that the approach evolved gradually and the author team acquired insights during the process, rather than following a predefined PPI plan. While a clearer initial plan might have enabled more extensive input sessions, such as interactive workshops, the flexible and evolving process reflects the realities and practical challenges of PPI, emphasizing the value of adapting as you learn. A next step could be to apply the approach to future research projects to help verify its usefulness and to provide opportunities for improvement. Applying the approach in future research projects may also help to broaden its applicability and relevance to other research fields.

In conclusion, the step-by-step approach presented is designed to be widely applicable across eHealth research and practically relevant for researchers working on eHealth projects. With appropriate adaptations, the approach could also support research in other fields. The approach was developed to address the gap between theory and practical guidance, and an apparent lack of well-organized, accessible resources. While there is no golden standard for implementing PPI, the goal of this approach is to ease the process of collaboration with end-users and inspire researchers to implement PPI into their own projects. This way, we can work towards valuable PPI in research and gain insight into how to implement it effectively in research.

## CRediT authorship contribution statement

MV: Conceptualization, Methodology, Project administration, Resources, Visualization, Writing - Original draft, Writing - Review and Editing.

RE: Methodology, Project administration, Resources, Writing - Original draft, Writing - Review and Editing.

CP: Conceptualization, Methodology, Project administration, Resources, Writing - Review and Editing.

JF: Methodology, Resources, Project administration, Writing - Review and Editing.

JA: Funding acquisition, Methodology, Resources, Supervision, Writing - Review and Editing.

EM: Conceptualization, Funding acquisition, Methodology, Resources, Supervision, Writing - Review and Editing.

AV: Conceptualization, Funding acquisition, Methodology, Resources, Supervision, Writing - Review and Editing.

## Declaration of Generative AI and AI-assisted technologies in the writing process

During the preparation of this work the author(s) used ChatGPT (OpenAI, 2025) in order to improve readability and language. After using this tool/service, the author(s) reviewed and edited the content as needed and take(s) full responsibility for the content of the publication.

## Funding

This work is part of three research projects:

*Digital asthma medication adherence intervention*

The Digital asthma medication adherence intervention study was supported by 10.13039/100004325AstraZeneca with an unrestricted grant.

*Data-supported treatment*

This work was funded by 10.13039/501100001826ZonMw as part of their Efficiency Research program (in Dutch: ‘Doelmatigheidsonderzoek’), under grant number 10390022210053.

*Perfect Fit*

The multidisciplinary research project Perfect Fit received funding from the Netherlands Organization for Scientific Research (NWO) program Commit2Data - Big Data & Health (project number 628.011.211). The program was funded by the following parties: NWO, the Netherlands Organization for Health Research and Development (ZonMw), Hartstichting, Ministry of Health, Welfare and Sport (VWS), Health Holland, and the Netherlands eScience Center. The publication reflects only the authors' views and the funders are not liable for any use that may be made of the information contained herein.

## Declaration of competing interest

The authors declare that they have no known competing financial interests or personal relationships that could have appeared to influence the work reported in this paper.

## Data Availability

Relevant resources and materials are included in the manuscript and its appendices. Additional documents (e.g., a sample collaboration agreement between researchers and end-users) are available from the corresponding author upon reasonable request.

## Supplementary data

Appendix A. Detailed reporting of patient and public involvement (PPI) using the Guidance for Reporting Involvement of Patients and the Public (GRIPP) 2 short form. Image 1

Appendix B. Worksheet step-by-step approach for researchers. Image 2

Appendix C. Werkblad stapsgewijze aanpak voor Nederlandse onderzoekers. Image 3

## Footnotes

2 https://www.involv.nl/.

3 https://research.longfonds.nl.
