# Supplementary material for: A practical step-by-step approach for patient and public involvement in eHealth intervention research: Lessons learned from three case projects
Source: Internet Interv. 2025 Dec 3;43:100896. doi: 10.1016/j.invent.2025.100896 (PMC12811673; doi:10.1016/j.invent.2025.100896)
Supplement: Supplementary file 1 — Appendix A. Detailed reporting of patient and public involvement (PPI) using the Guidance for Reporting Involvement of Patients and the Public (GRIPP) 2 short form. [file mmc1.docx]

**Appendix A.** *Detailed reporting of patient and public involvement (PPI) using the Guidance for Reporting Involvement of Patients and the Public (GRIPP) 2 short form*

| **Table A1**  *PPI in developing the step-by-step approach: Reporting according to the GRIPP2-SF (Staniszewska et al., 2017).* | |
| --- | --- |
| **Section and topic GRIPP2-SF** | **Description** |
| *1: Aim*  Report the aim. | Public health and health psychology researchers and end-users who had been long-term engaged in an eHealth intervention project (i.e., Perfect Fit project) were involved to refine and validate the step-by-step approach for implementing PPI in eHealth research. The aim of PPI in developing the approach was to improve the approach’s relevance, completeness, and practical applicability. |
| *2: Methods*  Provide a clear description of the methods used for PPI in the study. | The step-by-step approach was presented to other (eHealth) researchers to gather feedback and validate its content. This included two presentations at internal research meetings with colleagues from the author team and three presentations at (inter)national Health Psychology and Public Health conferences. These sessions allowed the researchers to assess whether the approach, recommendations, lessons learned, and resources were relevant, recognizable, and broadly applicable, and to identify any missing topics or additional insights.  In addition, in the Perfect Fit case study, individual end-evaluation interviews were conducted with the three members of the end-user advisory panel. The input gathered from these interviews was used to verify whether all relevant topics were included in the step-by-step approach and to determine whether the feedback aligned with recommendations and lessons learned, or whether revisions were required. Open-ended questions were designed around key topics from the approach, such as collaboration experiences, freedom to express opinions, perceptions of contribution to the project, and the evaluation of recognition and rewards. The questions were further informed by the Dutch version of the Public and Patient Engagement Evaluation Tool (PPEET; Bavelaar et al., 2021). |
| *3: Study Results*  Outcomes—Report the results of PPI in the study, including both positive and negative outcomes. | PPI contributed to the development of the step-by-step approach in several ways:   - Feedback from other (eHealth) researchers was overall positive and confirmed the relevance and broad applicability of the step-by-step approach. - Feedback led to specific improvements and additions, such as the inclusion of information on estimating necessary resources (e.g., budget) for meaningful PPI, in response to recurring questions from researchers. - The topic of PPI evaluation was also raised multiple times, prompting its more explicit inclusion in the approach. - The individual interviews with the Perfect Fit advisory panel members confirmed that the topics covered in the approach aligned well with their experiences. - A new lesson was added based on these interviews: the importance of providing ongoing and explicit feedback to end-users on how their input had been used in the project. |
| *4: Discussion and conclusions*  Outcomes—Comment on the extent to which PPI influenced the study overall. Describe positive and negative effects. | The PPI activities had a positive overall influence on the development of the step-by-step approach by enhancing its clarity, practical applicability, and alignment with the needs and expectations of both researchers and end-users. The PPI process facilitated the iterative integration of perspectives from both researchers and end-users, likely increasing the robustness and relevance of the final approach.  A factor that may have contributed to this positive influence was the evolving expertise of the end-users involved in the evaluation. While some of these end-users had little or no prior experience with PPI or research at the start of the project, their ongoing involvement over several years enabled them to develop valuable insights into research processes and PPI practices. This may have allowed them to reflect on the approach in a manner that was both accessible to other end-users and informed by practical experience.  However, there were also limitations. Opportunities to gather in-depth feedback from other researchers during conference presentations were limited due to time constraints inherent to such formats. As a result, potentially valuable critical input may not have been fully captured. Nonetheless, the approach was created, evaluated, and refined by the author team – researchers with diverse expertise and varying experiences in eHealth research and PPI. As such, researcher perspectives and reflections were incorporated not only through conference input, but also through the iterative development process within the author team itself. |
| *5: Reflections/critical perspective*  Comment critically on the study, reflecting on the things that went well and those that did not, so others can learn from this experience. | The PPI activities conducted to support the development of the approach yielded positive results. However, the process developed gradually and iteratively rather than following a predefined PPI plan. With a clearer plan from the outset, we might have organized more extensive input sessions, such as interactive workshops instead of standard conference presentations, to gather richer and more detailed feedback.  On the other hand, the iterative nature of the approach is logical and fitting for PPI. It emerged because we struggled to find practical guidance and relevant resources ourselves, which is why we believe that our approach will be relevant and useful for other researchers working on eHealth interventions.  Overall, while a more structured plan early on could have enhanced involvement, the flexible and evolving process reflects the realities and practical challenges of PPI, emphasizing the value of adapting as you learn. |
| *Note. PPI = Patient and Public Involvement* | |

**References**

Bavelaar, L., van Tol, L. S., Caljouw, M. A., & van der Steen, J. T. (2021). Nederlandse vertaling en eerste stappen in validatie van de PPEET om burger-en patiëntenparticipatie te evalueren. *TSG-Tijdschrift voor gezondheidswetenschappen*, *99*(4), 146-153.

Staniszewska, S., Brett, J., Simera, I., Seers, K., Mockford, C., Goodlad, S., Altman, D. G., Moher, D., Barber, R., Denegri, S., Entwistle, A., Littlejohns, P., Morris, C., Suleman, R., Thomas, V., & Tysall, C. (2017). GRIPP2 reporting checklists: tools to improve reporting of patient and public involvement in research. *Res Involv Engagem*, *3*, 13. <https://doi.org/10.1186/s40900-017-0062-2>
