# Supplementary material for: A practical step-by-step approach for patient and public involvement in eHealth intervention research: Lessons learned from three case projects
Source: Internet Interv. 2025 Dec 3;43:100896. doi: 10.1016/j.invent.2025.100896 (PMC12811673; doi:10.1016/j.invent.2025.100896)
Supplement: Supplementary file 2 — Appendix B. Worksheet step-by-step approach for researchers. [file mmc2.docx]

**Appendix B.** *Worksheet step-by-step approach for researchers*

This worksheet accompanies the step-by-step approach described in ‘A practical step-by-step approach for patient and public involvement in eHealth intervention research: Lessons learned from three case projects’.

It is intended for digital use. If you prefer to complete it on paper, we recommend enlarging the note fields in advance to ensure sufficient writing space.

**Worksheet Step-by-step Approach for Patient and Public Involvement**

Title of the project: …

Version: …

| General PPI resources |
| --- |
| [Engagement in Research](https://www.pcori.org/engagement-research/value-engagement-research) (Patient-centered Outcomes Research Institute, 2025) |
| [Kickstarter for researchers](https://www.involv.nl/advies-voor-onderzoekers/kickstarter-for-researchers-en) (INVOLV, n.d.) |
| [CeHReS Roadmap 2.0](https://doi.org/10.2196/59601) (Kip et al., 2025) |
| [Interactive RE-AIM Planning Tool](https://re-aim.org/applying-the-re-aim-framework/re-aim-guidance/use-when-planning-a-project/planning-tool/) (RE-AIM, 2025) |

**Step 1: WHERE**

Where in the eHealth evaluation cycle (Bonten et al., 2020) is your research project positioned?

| *Notes:* |
| --- |

| Recommendations | Resources |
| --- | --- |
| Identify the research phase to guide PPI planning | [eHealth evaluation cycle](https://doi.org/10.2196/17774) (Bonten et al., 2020) and [eHealth methodology guide](https://citrienfonds-ehealth.nl/e-health-toolkit/onderzoek/e-health-evaluation-methodology/overview-of-methods) (Citrienfonds, n.d.) |
|  | [Participatory action research](https://doi.org/10.1136/jech.2004.028662) (Baum et al., 2006) |
| Involve end-users early | [eHealth evaluation cycle](https://doi.org/10.2196/17774) (Bonten et al., 2020) and [eHealth methodology guide](https://citrienfonds-ehealth.nl/e-health-toolkit/onderzoek/e-health-evaluation-methodology/overview-of-methods) (Citrienfonds, n.d.) |

**Step 2 and 3: WHY & WHO**

Why do you want to use PPI? Who is your target population?

| *Reflective questions WHY:*   - *Why do you want to use PPI in your research project?* - *What are the overall aims that you would like to achieve?* - *What could be the added benefit of using PPI in your project?* - *How do your PPI aims align with the broader objectives of your study?*   *Reflective questions WHO:*   - *Who is your target population?* - *Who would benefit from your research?* - *Who do you need to achieve your PPI aims?* - *Who can represent the population of your research project?* - *What lived experiences or perspectives are essential to achieving your PPI aims?* - *Are there groups that are often underrepresented but whose input is critical for your project?*   *Notes:* |
| --- |

| Recommendations | Resources |
| --- | --- |
| Set realistic goals for PPI | [Structuring involvement](https://www.involv.nl/advies-voor-onderzoekers/kickstarter/structuring-involvement-en) (INVOLV, n.d.) |
| Ensure representative and inclusive PPI | [Representative involvement](https://www.pcori.org/engagement-research/engagement-resources/foundational-expectations/representative-involvement) (Patient-centered Outcomes Research Institute, 2025) |
|  | [Design Kit](https://www.designkit.org/methods/define-your-audience.html) (IDEO.org, n.d.) |
| Match end-users to your PPI aims | [Representative involvement](https://www.pcori.org/engagement-research/engagement-resources/foundational-expectations/representative-involvement) (Patient-centered Outcomes Research Institute, 2025) |
|  | [Matching researchers’ needs and patients’ contributions: practical tips for meaningful patient engagement from the field of rheumatology](https://ard.bmj.com/content/82/3/312) (Schoemaker et al., 2023) |
| Collaborate with patient organizations | Appendix ‘How to Find Public Contributors.’ from: (Bagley et al., 2016) |

**Step 4: HOW**

How are you going to achieve your aims?

**A)** Timing (research phase) and participation role

| *Reflective questions:*   - *When will you involve your end-users?* - *Which decisions or stages in your project would benefit from end-user input?* - *Do you need continuity in involvement (e.g., an advisory panel) or one-time involvement?* - *In what ways can you involve them?* - *What involvement role is desirable and achievable for end-users in each activity?*   *Notes:* |
| --- |

| Recommendations | Resources |
| --- | --- |
| Plan participatory activities per research phase | [The Involvement Matrix](https://www.kcrutrecht.nl/involvement-matrix/) (Kenniscentrum Revalidatiegeneeskunde Utrecht, 2019) |
|  | [Creating a Visual Map of the Study Activity Guide With Worksheet](https://research-teams.pcori.org/sites/default/files/2021-03/CAVMOTS-ActivityGuide-508.pdf) (Patient-centered Outcomes Research Institute, 2025) |
| Define roles per activity together with end-users | [The Involvement Matrix](https://www.kcrutrecht.nl/involvement-matrix/) (Kenniscentrum Revalidatiegeneeskunde Utrecht, 2019) |
|  | [Meaningful Inclusion of Partners in Decision Making](https://www.pcori.org/engagement-research/engagement-resources/foundational-expectations/meaningful-inclusion) (Patient-centered Outcomes Research Institute, 2025) |
|  | [Defining roles and responsibilities](https://research-teams.pcori.org/best-practices#Defining%20Roles%20and%20Responsibilities) (Patient-centered Outcomes Research Institute, 2025) |
|  | [Matching Strengths to Research Tasks (Activity Guide With Worksheet)](https://research-teams.pcori.org/sites/default/files/2021-03/MSTRT-ActivityGuide-508_1.pdf) (Patient-centered Outcomes Research Institute, 2025) |

**B)** Participatory methods

| *Reflective questions:*   - *How will you recruit the people you need?* - *How will you capture their voice?* - *Will they speak from personal experience or as representatives?* - *Which participatory tools and methods are most appropriate?* - *How accessible are these methods to the end-users you intend to involve?*   *Notes:* |
| --- |

| Recommendations | Resources |
| --- | --- |
| Align expectations through introductory meetings | [The Involvement Matrix](https://www.kcrutrecht.nl/involvement-matrix/) (Kenniscentrum Revalidatiegeneeskunde Utrecht, 2019) |
|  | [Build Capacity to Work as a Team](https://www.pcori.org/engagement-research/engagement-resources/foundational-expectations/build-capacity) (Patient-centered Outcomes Research Institute, 2025) |
| Clarify whose voice is represented | [Matching researchers’ needs and patients’ contributions: practical tips for meaningful patient engagement from the field of rheumatology](https://ard.bmj.com/content/82/3/312) (Schoemaker et al., 2023) |
| Tailor meeting formats to end-user needs | [Foundational framework summarizing principles and best practice activities supporting patient stakeholder engagement in research](https://onlinelibrary.wiley.com/doi/10.1111/hex.12873) (Harrison et al., 2019) |
|  | [Build Capacity to Work as a Team](https://www.pcori.org/engagement-research/engagement-resources/foundational-expectations/build-capacity) (Patient-centered Outcomes Research Institute, 2025) |
| Use creative methods to surface implicit needs | [Research through design](https://www.interaction-design.org/literature/book/the-encyclopedia-of-human-computer-interaction-2nd-ed/research-through-design) (Stappers & Giaccardi, 2017) |
|  | [Methods Used in Co-Creation Within the Health CASCADE Co-Creation Database and Gray Literature: Systematic Methods Overview](https://www.i-jmr.org/2024/1/e59772) (Agnello et al., 2024) |
| Step into the world of your end-users | [Change by Design](https://doi.org/10.1111/j.1540-5885.2011.00806.x) (Brown & Katz, 2011) |

**Step 5: WHAT**

What considerations and conditions need to be taken into account to facilitate PPI?

**A)** Considerations and context of the research project

| *Reflective questions:*   - *What characteristics of the research project should be considered when planning and implementing PPI?* - *What is your timeline and available budget?* - *How is your research team composed, and who will be responsible for organizing and facilitating PPI?* - *How will internal communication and feedback loops be established to share and act on PPI insights?* - *Are there other stakeholders whose input or approval is necessary (e.g., software developers)?* - *Are there important collaborations with other parties that may affect PPI?*   *Notes:* |
| --- |

| Recommendations | Resources |
| --- | --- |
| Adapt participatory activities to context and goals | [Build Capacity to Work as a Team](https://www.pcori.org/engagement-research/engagement-resources/foundational-expectations/build-capacity) (Patient-centered Outcomes Research Institute, 2025) |
| Coordinate communication and feedback | [Meaningful Inclusion of Partners in Decision Making](https://www.pcori.org/engagement-research/engagement-resources/foundational-expectations/meaningful-inclusion) (Patient-centered Outcomes Research Institute, 2025) |
| Plan resources and budget for meaningful PPI | [Patient stakeholder engagement in research: A narrative review to describe foundational principles and best practice activities](https://onlinelibrary.wiley.com/doi/10.1111/hex.12873) (Harrison et al., 2019) |
|  | [Plan internal processes so that payment occurs in a timely and efficient manner](https://www.pcori.org/engagement-research/engagement-resources/foundational-expectations/dedicated-funds-engagement-partner-compensation#section_plan_for_timely_compensation) (Patient-centered Outcomes Research Institute, 2025) |

**B)** Conditions for successful collaboration

| *Reflective questions:*   - *What is expected from each stakeholder involved in PPI?* - *What are the roles, rights, and responsibilities of researchers and end-users?* - *What conditions are necessary to enable a fruitful collaboration?* - *What practical arrangements are required (like logistics, budget, and compensation)?* - *What kind of support or resources do end-users need to participate meaningfully?* - *What strategies can you use to build trust and maintain engagement over time?*   *Notes:* |
| --- |

| Recommendations | Resources |
| --- | --- |
| Discuss roles, expectations, and limitations | Template for agreement (designed by C. C. Poot; see ‘Availability of data and materials’ in main text) |
|  | [Patient stakeholder engagement in research: A narrative review to describe foundational principles and best practice activities](https://onlinelibrary.wiley.com/doi/10.1111/hex.12873) (Harrison et al., 2019) |
| Ensure openness, trust, and adaptive collaboration | [Patient stakeholder engagement in research: A narrative review to describe foundational principles and best practice activities](https://onlinelibrary.wiley.com/doi/10.1111/hex.12873) (Harrison et al., 2019) |
|  | [Meaningful Inclusion of Partners in Decision Making](https://www.pcori.org/engagement-research/engagement-resources/foundational-expectations/meaningful-inclusion) (Patient-centered Outcomes Research Institute, 2025) |
| Enable informed and confident participation | [Patient stakeholder engagement in research: A narrative review to describe foundational principles and best practice activities](https://onlinelibrary.wiley.com/doi/10.1111/hex.12873) (Harrison et al., 2019) |
|  | [Build Capacity to Work as a Team](https://www.pcori.org/engagement-research/engagement-resources/foundational-expectations/build-capacity) (Patient-centered Outcomes Research Institute, 2025) |
|  | [Research Fundamentals: Preparing You to Successfully Contribute to Research](https://www.pcori.org/engagement-research/engagement-resources/research-fundamentals-preparing-you-successfully-contribute-research) (Patient-centered Outcomes Research Institute, 2025) |
| Give concrete and timely feedback on input | [Structuring involvement](https://www.involv.nl/advies-voor-onderzoekers/kickstarter/structuring-involvement-en) (INVOLV, n.d.) |
|  | [Patient stakeholder engagement in research: A narrative review to describe foundational principles and best practice activities](https://onlinelibrary.wiley.com/doi/10.1111/hex.12873) (Harrison et al., 2019) |
|  | [Meaningful Inclusion of Partners in Decision Making](https://www.pcori.org/engagement-research/engagement-resources/foundational-expectations/meaningful-inclusion) (Patient-centered Outcomes Research Institute, 2025) |
| Value, acknowledge, and compensate end-users | [Patient stakeholder engagement in research: A narrative review to describe foundational principles and best practice activities](https://onlinelibrary.wiley.com/doi/10.1111/hex.12873) (Harrison et al., 2019) |
|  | [Dedicated Funds for Engagement & Partner Compensation](https://www.pcori.org/engagement-research/engagement-resources/foundational-expectations/dedicated-funds-engagement-partner-compensation) (Patient-centered Outcomes Research Institute, 2025) |
|  | [Guidance on authorship with and acknowledgement of patient partners in patient-oriented research](https://link.springer.com/article/10.1186/s40900-020-00213-6) (Richards et al., 2020) |
| Support sustainable collaboration | [Patient stakeholder engagement in research: A narrative review to describe foundational principles and best practice activities](https://onlinelibrary.wiley.com/doi/10.1111/hex.12873) (Harrison et al., 2019) |
|  | [Meaningful Inclusion of Partners in Decision Making](https://www.pcori.org/engagement-research/engagement-resources/foundational-expectations/meaningful-inclusion) (Patient-centered Outcomes Research Institute, 2025) |

**Step 6: EVALUATION**

*Intermediate and end of project evaluation*

How did the PPI process unfold?

| *Reflective questions:*   - *What aspects are important to evaluate for both you and the end-users?* - *How is the PPI process progressing?* - *Are the predefined PPI aims being met?* - *Does it align with everyone’s expectations and needs?* - *Are there areas for improvement or elements that should be maintained?* - *What has been the perceived added value of PPI so far?* - *How can the outcomes or relationships from this collaboration be sustained?*   *Notes:* |
| --- |

| Recommendations | Resources |
| --- | --- |
| Integrate ongoing monitoring and evaluation | [Patient stakeholder engagement in research: A narrative review to describe foundational principles and best practice activities](https://onlinelibrary.wiley.com/doi/10.1111/hex.12873) (Harrison et al., 2019) |
|  | [Ongoing Review & Assessment of Engagement](https://www.pcori.org/engagement-research/engagement-resources/foundational-expectations/ongoing-review-and-assessment) (Patient-centered Outcomes Research Institute, 2025) |
|  | [Patient resources](https://ossu.ca/for-patients/resources/) (Ontario SPOR SUPPORT Unit, n.d.) |
| Conduct an end-of-project evaluation | [PPEET questionnaire](https://doi.org/10.1111/hex.12378) (Abelson et al., 2016) |
|  | [An Empirical‐Theoretical Analysis Framework for Public Participation in Environmental Impact Assessment](https://www.tandfonline.com/doi/abs/10.1080/713676582) (Palerm, 2000) |
|  | [A model and measure for quality service user involvement in health research](https://doi.org/10.1111/j.1470-6431.2010.00901.x) (Morrow et al., 2010) |
|  | [Patient stakeholder engagement in research: A narrative review to describe foundational principles and best practice activities](https://onlinelibrary.wiley.com/doi/10.1111/hex.12873) (Harrison et al., 2019) |
|  | [Patient resources](https://ossu.ca/for-patients/resources/) (Ontario SPOR SUPPORT Unit, n.d.) |
| Carry forward knowledge and collaboration | [Community-Based Participatory Research](https://doi.org/10.1037/amp0000167)  (e.g., Collins et al., 2018) |
|  | [Participatory action research](https://doi.org/10.1136/jech.2004.028662) (Baum et al., 2006) |

**References**

Abelson, J., Li, K., Wilson, G., Shields, K., Schneider, C., & Boesveld, S. (2016). Supporting quality public and patient engagement in health system organizations: development and usability testing of the Public and Patient Engagement Evaluation Tool. *Health Expectations*, *19*(4), 817–827. <https://doi.org/10.1111/hex.12378>

Agnello, D. M., Balaskas, G., Steiner, A., & Chastin, S. (2024). Methods Used in Co-Creation Within the Health CASCADE Co-Creation Database and Gray Literature: Systematic Methods Overview. *Interactive Journal of Medical Research*, *13*. <https://doi.org/10.2196/59772>

Bagley, H. J., Short, H., Harman, N. L., Hickey, H. R., Gamble, C. L., Woolfall, K., Young, B., & Williamson, P. R. (2016). A patient and public involvement (PPI) toolkit for meaningful and flexible involvement in clinical trials - a work in progress. *Res Involv Engagem*, *2*, 15. <https://doi.org/10.1186/s40900-016-0029-8>

Baum, F., MacDougall, C., & Smith, D. (2006). Participatory action research. *J Epidemiol Community Health*, *60*(10), 854–857. <https://doi.org/10.1136/jech.2004.028662>

Bonten, T. N., Rauwerdink, A., Wyatt, J. C., Kasteleyn, M. J., Witkamp, L., Riper, H., van Gemert-Pijnen, L. J., Cresswell, K., Sheikh, A., Schijven, M. P., Chavannes, N. H., & Group, E. H. E. R. (2020). Online Guide for Electronic Health Evaluation Approaches: Systematic Scoping Review and Concept Mapping Study. *J Med Internet Res*, *22*(8), e17774. <https://doi.org/10.2196/17774>

Brown, T., & Katz, B. (2011). Change by Design. *Journal of Product Innovation Management*, *28*(3). <https://doi.org/10.1111/j.1540-5885.2011.00806.x>

Citrienfonds. (n.d.). *eHealth methodology guide*. Retrieved August 2, 2025 from <https://citrienfonds-ehealth.nl/e-health-toolkit/onderzoek/e-health-evaluation-methodology/overview-of-methods/>

Collins, S. E., Clifasefi, S. L., Stanton, J., Straits, K. J. E., Gil-Kashiwabara, E., Espinosa, P. R., Nicasio, A. V., Andrasik, M. P., Hawes, S. M., Miller, K. A., Nelson, L. A., Orfaly, V. E., Duran, B. M., Wallerstein, N., & Board, L. A. (2018). Community-Based Participatory Research (CBPR): Towards Equitable Involvement of Community in Psychology Research. *American Psychologist*, *73*(7), 884–898. <https://doi.org/10.1037/amp0000167>

Harrison, J. D., Auerbach, A. D., Anderson, W., Fagan, M., Carnie, M., Hanson, C., Banta, J., Symczak, G., Robinson, E., Schnipper, J., Wong, C., & Weiss, R. (2019). Patient stakeholder engagement in research: A narrative review to describe foundational principles and best practice activities. *Health Expect*, *22*(3), 307–316. <https://doi.org/10.1111/hex.12873>

IDEO.org. (n.d.). *Define your audience*. Retrieved August 12, 2025 from <https://www.designkit.org/methods/define-your-audience.html>

INVOLV. (n.d.). *Kickstarter for researchers*. Retrieved June 2, 2025 from <https://www.involv.nl/advies-voor-onderzoekers/kickstarter-for-researchers-en>

Kenniscentrum Revalidatiegeneeskunde Utrecht. (2019). *Involvement Matrix*. Kenniscentrum Revalidatiegeneeskunde Utrecht. Retrieved 3 February from <https://www.kcrutrecht.nl/involvement-matrix/>

Kip, H., Beerlage-de Jong, N., van Gemert-Pijnen, L., & Kelders, S. M. (2025). The CeHRes Roadmap 2.0: Update of a Holistic Framework for Development, Implementation, and Evaluation of eHealth Technologies. *J Med Internet Res*, *27*(1), e59601. <https://doi.org/10.2196/59601>

Morrow, E., Ross, F., Grocott, P., & Bennett, J. (2010). A model and measure for quality service user involvement in health research. *International Journal Of Consumer Studies*, *34*(5). <https://doi.org/10.1111/j.1470-6431.2010.00901.x>

Ontario SPOR SUPPORT Unit. (n.d.). *Patient resources*. Retrieved August 12, 2025 from <https://ossu.ca/for-patients/resources/>

Palerm, J. R. (2000). An Empirical‐Theoretical Analysis Framework for Public Participation in Environmental Impact Assessment. *Journal of Environmental Planning and Management*, *43*(5). <https://doi.org/10.1080/713676582>

Patient-centered Outcomes Research Institute. (2025). *Engagement in Research*. Patient-centered Outcomes Research Institute. Retrieved 3 February from <https://www.pcori.org/engagement-research/value-engagement-research>

RE-AIM. (2025). *Interactive RE-AIM Planning Tool*. Retrieved August 4, 2025 from <https://re-aim.org/applying-the-re-aim-framework/re-aim-guidance/use-when-planning-a-project/planning-tool/>

Richards, D. P., Birnie, K. A., Eubanks, K., Lane, T., Linkiewich, D., Singer, L., Stinson, J. N., & Begley, K. N. (2020). Guidance on authorship with and acknowledgement of patient partners in patient-oriented research. *Res Involv Engagem*, *6*, 38. <https://doi.org/10.1186/s40900-020-00213-6>

Schoemaker, C. G., Richards, D. P., & de Wit, M. (2023). Matching researchers' needs and patients' contributions: practical tips for meaningful patient engagement from the field of rheumatology. *Annals of the Rheumatic Diseases*, *82*(3), 312–315. <https://doi.org/10.1136/ard-2022-223561>

Stappers, P., & Giaccardi, E. (2017). *Research through Design*. Soegaard, M. & Friis-Dam, R. Retrieved August 12, 2025 from <https://www.interaction-design.org/literature/book/the-encyclopedia-of-human-computer-interaction-2nd-ed/research-through-design>
