# Supplementary material for: A practical step-by-step approach for patient and public involvement in eHealth intervention research: Lessons learned from three case projects
Source: Internet Interv. 2025 Dec 3;43:100896. doi: 10.1016/j.invent.2025.100896 (PMC12811673; doi:10.1016/j.invent.2025.100896)
Supplement: Supplementary file 3 — Appendix C. Werkblad stapsgewijze aanpak voor Nederlandse onderzoekers. [file mmc3.docx]

**Appendix C.** *Werkblad stapsgewijze aanpak voor Nederlandse onderzoekers*

Dit werkblad hoort bij het stappenplan zoals beschreven in ‘A practical step-by-step approach for patient and public involvement in eHealth intervention research: Lessons learned from three case projects’.

Het werkblad is bedoeld voor digitaal gebruik. Indien je het liever op papier invult, raden we aan om de notitievakken vooraf te vergroten zodat er voldoende schrijfruimte is.

**Werkblad voor stapsgewijze aanpak voor (patiënt)participatie**

Naam van het project: …

Versie: …

| Algemene PPI hulpmiddelen |
| --- |
| [Engagement in Research](https://www.pcori.org/engagement-research/value-engagement-research) (Patient-centered Outcomes Research Institute, 2025) |
| [Kickstarter voor onderzoekers](https://www.involv.nl/advies-voor-onderzoekers/kickstarter) (INVOLV, n.d.–a) |
| [CeHReS Roadmap 2.0](https://doi.org/10.2196/59601) (Kip et al., 2025) |
| [Interactive RE-AIM Planning Tool](https://re-aim.org/applying-the-re-aim-framework/re-aim-guidance/use-when-planning-a-project/planning-tool/) (RE-AIM, 2025) |

**Stap 1: WAAR**

Waar in de eHealth evaluatiecyclus (Bonten et al., 2020) bevindt je onderzoeksproject zich?

| *Notities:* |
| --- |

| Aanbevelingen | Hulpmiddelen |
| --- | --- |
| Bepaal de onderzoeksfase om de planning van PPI daarop af te stemmen | [eHealth evaluatiecyclus](https://doi.org/10.2196/17774) (Bonten et al., 2020) en [eHealth methodology guide](https://citrienfonds-ehealth.nl/e-health-toolkit/onderzoek/e-health-evaluation-methodology/overview-of-methods) (Citrienfonds, n.d.) |
|  | [Participatory action research](https://doi.org/10.1136/jech.2004.028662) (Baum et al., 2006) |
| Betrek eindgebruikers in een vroeg stadium | [eHealth evaluatiecyclus](https://doi.org/10.2196/17774) (Bonten et al., 2020) en [eHealth methodology guide](https://citrienfonds-ehealth.nl/e-health-toolkit/onderzoek/e-health-evaluation-methodology/overview-of-methods) (Citrienfonds, n.d.) |
|  | [De inclusieve eHealth Handreiking – Ontwikkelen met de doelgroep](https://www.tudelft.nl/inclusive-ehealth-guide/ontwikkelen/ontwikkelen-met-de-doelgroep) (Faber et al., 2023) |

**Stap 2 en 3: WAAROM & WIE**

Waarom wil je (patiënt)participatie toepassen? Wie is je doelgroep?

| *Vragen ter overweging WAAROM:*   - *Waarom wil je (patiënt)participatie toepassen in je onderzoeksproject?* - *Wat zijn de algemene doelen die je wilt bereiken?* - *Wat zou de toegevoegde waarde zijn van (patiënt)participatie in je project?* - *Hoe sluiten je participatie doelen aan bij de bredere doelstellingen van je onderzoek?*   *Vragen ter overweging WIE:*   - *Wie is je doelgroep?* - *Wie heeft er baat bij jouw onderzoek?* - *Wie heb je nodig om je participatie doelen te bereiken?* - *Wie kan de populatie van je onderzoeksproject vertegenwoordigen?* - *Welke ervaringen of perspectieven zijn essentieel voor het bereiken van je participatie doelen?* - *Zijn er groepen die vaak ondervertegenwoordigd zijn, maar wiens inbreng cruciaal is voor je project?*   *Notities:* |
| --- |

| Aanbevelingen | Hulpmiddelen |
| --- | --- |
| Stel realistische doelen voor participatie | [Participatie vormgeven](https://www.involv.nl/advies-voor-onderzoekers/kickstarter/participatie-vormgeven) (INVOLV, n.d.–a) |
|  | [Patiëntenparticipatie in Clinical Trial Roadmap](https://participatiekompas.nl/clinical-trial-roadmap) (Dutch Oncology Research Platform, 2023) |
| Zorg voor representatieve en inclusieve participatie | [Representative involvement](https://www.pcori.org/engagement-research/engagement-resources/foundational-expectations/representative-involvement) (Patient-centered Outcomes Research Institute, 2025) |
|  | [Design Kit](https://www.designkit.org/methods/define-your-audience.html) (IDEO.org, n.d.) |
| Koppel eindgebruikers aan je participatie doelen | [Representative involvement](https://www.pcori.org/engagement-research/engagement-resources/foundational-expectations/representative-involvement) (Patient-centered Outcomes Research Institute, 2025) |
|  | [Matching researchers’ needs and patients’ contributions: practical tips for meaningful patient engagement from the field of rheumatology](https://ard.bmj.com/content/82/3/312) (Schoemaker et al., 2023) |
| Werk samen met patiëntenorganisaties | Appendix ‘How to Find Public Contributors.’ from: (Bagley et al., 2016) |
|  | [Vinden van patiëntenvereniging](https://kennisbank.patientenfederatie.nl/app/answers/detail/a_id/29/~/vinden-van-pati%C3%ABntenvereniging) (Patiëntenfederatie Nederland, 2025) |

**Stap 4: HOE**

Hoe ga je je doelen bereiken?

**A)** Timing (onderzoeksfase) en participatie rol

| *Vragen ter overweging:*   - *Wanneer betrek je eindgebruikers erbij?* - *Welke beslissingen of fasen in je project zouden baat hebben bij de inbreng van eindgebruikers?* - *Heb je continuïteit in betrokkenheid nodig (bijv. een adviespanel) of eenmalige betrokkenheid?* - *Op welke manieren kun je ze betrekken?* - *Welke rol is wenselijk en haalbaar voor eindgebruikers bij elke activiteit?*   *Notities:* |
| --- |

| Aanbevelingen | Hulpmiddelen |
| --- | --- |
| Plan participatie activiteiten per onderzoeksfase | [De participatiematrix](https://www.kcrutrecht.nl/producten/participatiematrix/) (Kenniscentrum Revalidatiegeneeskunde Utrecht, 2019) |
|  | [Creating a Visual Map of the Study Activity Guide With Worksheet](https://research-teams.pcori.org/sites/default/files/2021-03/CAVMOTS-ActivityGuide-508.pdf) (Patient-centered Outcomes Research Institute, 2025) |
|  | [Patiëntenparticipatie in Clinical Trial Roadmap](https://participatiekompas.nl/clinical-trial-roadmap) (Dutch Oncology Research Platform, 2023) |
| Bepaal de rollen per activiteit samen met eindgebruikers | [De participatiematrix](https://www.kcrutrecht.nl/producten/participatiematrix/) (Kenniscentrum Revalidatiegeneeskunde Utrecht, 2019) |
|  | [Rollen in participatie](https://www.involv.nl/trainingen/rollen-participatie-online-module) (INVOLV, n.d.–b) |
|  | [Meaningful Inclusion of Partners in Decision Making](https://www.pcori.org/engagement-research/engagement-resources/foundational-expectations/meaningful-inclusion) (Patient-centered Outcomes Research Institute, 2025) |
|  | [Defining roles and responsibilities](https://research-teams.pcori.org/best-practices#Defining%20Roles%20and%20Responsibilities) (Patient-centered Outcomes Research Institute, 2025) |
|  | [Matching Strengths to Research Tasks (Activity Guide With Worksheet)](https://research-teams.pcori.org/sites/default/files/2021-03/MSTRT-ActivityGuide-508_1.pdf) (Patient-centered Outcomes Research Institute, 2025) |

**B)** Participatie methoden

| *Vragen ter overweging:*   - *Hoe werf je de mensen die je nodig hebt?* - *Hoe ga je hun mening in beeld brengen?* - *Zullen ze spreken uit persoonlijke ervaring of als vertegenwoordigers?* - *Welke participatieve hulpmiddelen en methoden zijn het meest geschikt?* - *Hoe toegankelijk zijn deze methoden voor de eindgebruikers die je erbij wilt betrekken?*   *Notities:* |
| --- |

| Aanbevelingen | Hulpmiddelen |
| --- | --- |
| Stem verwachtingen af via kennismakingsbijeenkomsten | [De participatiematrix](https://www.kcrutrecht.nl/producten/participatiematrix/) (Kenniscentrum Revalidatiegeneeskunde Utrecht, 2019) |
|  | [Build Capacity to Work as a Team](https://www.pcori.org/engagement-research/engagement-resources/foundational-expectations/build-capacity) (Patient-centered Outcomes Research Institute, 2025) |
| Verduidelijk wiens stem wordt vertegenwoordigd | [Matching researchers’ needs and patients’ contributions: practical tips for meaningful patient engagement from the field of rheumatology](https://ard.bmj.com/content/82/3/312) (Schoemaker et al., 2023) |
| Pas vergaderformats aan op de behoeften van eindgebruikers | [Foundational framework summarizing principles and best practice activities supporting patient stakeholder engagement in research](https://onlinelibrary.wiley.com/doi/10.1111/hex.12873) (Harrison et al., 2019) |
|  | [Build Capacity to Work as a Team](https://www.pcori.org/engagement-research/engagement-resources/foundational-expectations/build-capacity) (Patient-centered Outcomes Research Institute, 2025) |
| Gebruik creatieve methoden om impliciete behoeften aan het licht te brengen | [100 werkvormen voor docenten en trainers](https://www.icm.nl/extra/100-werkvormen-docenten-en-trainers/) (ICM Opleidingen & Trainingen, n.d.) of [Activerende werkvormen](https://teaching-and-learning-collection.sites.uu.nl/knowledge_item/activerende-werkvormen/) (Torfs & Wismans, 2015) |
|  | [Research through design](https://www.interaction-design.org/literature/book/the-encyclopedia-of-human-computer-interaction-2nd-ed/research-through-design) (Stappers & Giaccardi, 2017) |
|  | [Methods Used in Co-Creation Within the Health CASCADE Co-Creation Database and Gray Literature: Systematic Methods Overview](https://www.i-jmr.org/2024/1/e59772) (Agnello et al., 2024) |
| Stap in de wereld van je eindgebruikers | [Change by Design](https://doi.org/10.1111/j.1540-5885.2011.00806.x) (Brown & Katz, 2011) |

**Stap 5: WAT**

Met welke overwegingen en voorwaarden moet rekening worden gehouden om (patiënt)participatie mogelijk te maken?

**A)** Overwegingen en context van het onderzoeksproject

| *Vragen ter overweging:*   - *Met welke kenmerken van het onderzoeksproject moet rekening worden gehouden bij het plannen en implementeren van participatie?* - *Wat is je tijdlijn en beschikbare budget?* - *Hoe is je onderzoeksteam samengesteld, en wie wordt verantwoordelijk voor het organiseren en begeleiden van participatie?* - *Hoe worden interne communicatie- en feedbackloops ingericht om participatie inzichten te delen en toe te passen?* - *Zijn er andere belanghebbenden wiens inbreng of goedkeuring nodig is (bijv. softwareontwikkelaars)?* - *Zijn er belangrijke samenwerkingsverbanden met andere partijen die participatie kunnen beïnvloeden?*   *Notities:* |
| --- |

| Aanbevelingen | Hulpmiddelen |
| --- | --- |
| Pas participatie activiteiten aan op de context en doelen | [Build Capacity to Work as a Team](https://www.pcori.org/engagement-research/engagement-resources/foundational-expectations/build-capacity) (Patient-centered Outcomes Research Institute, 2025) |
|  | [Ontwikkelen met de doelgroep](https://www.tudelft.nl/inclusive-ehealth-guide/ontwikkelen/ontwikkelen-met-de-doelgroep) (Faber et al., 2023) |
|  | [eHealth of digitale zorg die iedereen kan gebruiken: hoe doe je dat?](https://www.pharos.nl/ehealth-en-digitale-zorg-voor-iedereen/) (Pharos, n.d.) |
| Coördineer communicatie en feedback | [Meaningful Inclusion of Partners in Decision Making](https://www.pcori.org/engagement-research/engagement-resources/foundational-expectations/meaningful-inclusion) (Patient-centered Outcomes Research Institute, 2025) |
| Zorg voor voldoende middelen en budget voor zinvolle participatie | [Patient stakeholder engagement in research: A narrative review to describe foundational principles and best practice activities](https://onlinelibrary.wiley.com/doi/10.1111/hex.12873) (Harrison et al., 2019) |
|  | [Plan internal processes so that payment occurs in a timely and efficient manner](https://www.pcori.org/engagement-research/engagement-resources/foundational-expectations/dedicated-funds-engagement-partner-compensation#section_plan_for_timely_compensation) (Patient-centered Outcomes Research Institute, 2025) |

**B)** Voorwaarden voor geslaagde samenwerking

| *Vragen ter overweging:*   - *Wat wordt er verwacht van elke belanghebbende die betrokken is bij participatie?* - *Wat zijn de rollen, rechten, en verantwoordelijkheden van onderzoekers en eindgebruikers?* - *Welke voorwaarden zijn nodig om een productieve samenwerking mogelijk te maken?* - *Welke praktische regelingen zijn nodig (zoals logistiek, budget en compensatie)?* - *Wat voor ondersteuning of hulpmiddelen hebben eindgebruikers nodig voor zinvolle participatie?* - *Welke strategieën kun je gebruiken om vertrouwen op te bouwen en betrokkenheid te behouden?*   *Notities:* |
| --- |

| Aanbevelingen | Hulpmiddelen |
| --- | --- |
| Bespreek rollen, verwachtingen en beperkingen | Voorbeeld overeenkomst (ontworpen door C. C. Poot; zie ‘Availability of data and materials’ in de hoofdtekst) |
|  | [Patient stakeholder engagement in research: A narrative review to describe foundational principles and best practice activities](https://onlinelibrary.wiley.com/doi/10.1111/hex.12873) (Harrison et al., 2019) |
| Zorg voor openheid, vertrouwen en flexibele samenwerking | [Patient stakeholder engagement in research: A narrative review to describe foundational principles and best practice activities](https://onlinelibrary.wiley.com/doi/10.1111/hex.12873) (Harrison et al., 2019) |
|  | [Meaningful Inclusion of Partners in Decision Making](https://www.pcori.org/engagement-research/engagement-resources/foundational-expectations/meaningful-inclusion) (Patient-centered Outcomes Research Institute, 2025) |
| Maak geïnformeerde en zelfverzekerde deelname mogelijk | [Patient stakeholder engagement in research: A narrative review to describe foundational principles and best practice activities](https://onlinelibrary.wiley.com/doi/10.1111/hex.12873) (Harrison et al., 2019) |
|  | [Build Capacity to Work as a Team](https://www.pcori.org/engagement-research/engagement-resources/foundational-expectations/build-capacity) (Patient-centered Outcomes Research Institute, 2025) |
|  | [Research Fundamentals: Preparing You to Successfully Contribute to Research](https://www.pcori.org/engagement-research/engagement-resources/research-fundamentals-preparing-you-successfully-contribute-research) (Patient-centered Outcomes Research Institute, 2025) |
|  | [Training 'INVOLV'](https://www.involv.nl/trainingen/trainingenoverzicht?page=4) (INVOLV, n.d.–c) |
| Geef concrete en tijdige feedback op inbreng | [Participatie vormgeven](https://www.involv.nl/advies-voor-onderzoekers/kickstarter/participatie-vormgeven) (INVOLV, n.d.–a) |
|  | [Patient stakeholder engagement in research: A narrative review to describe foundational principles and best practice activities](https://onlinelibrary.wiley.com/doi/10.1111/hex.12873) (Harrison et al., 2019) |
|  | [Meaningful Inclusion of Partners in Decision Making](https://www.pcori.org/engagement-research/engagement-resources/foundational-expectations/meaningful-inclusion) (Patient-centered Outcomes Research Institute, 2025) |
| Waardeer, erken en vergoed eindgebruikers | [Patient stakeholder engagement in research: A narrative review to describe foundational principles and best practice activities](https://onlinelibrary.wiley.com/doi/10.1111/hex.12873) (Harrison et al., 2019) |
|  | [Dedicated Funds for Engagement & Partner Compensation](https://www.pcori.org/engagement-research/engagement-resources/foundational-expectations/dedicated-funds-engagement-partner-compensation) (Patient-centered Outcomes Research Institute, 2025) |
|  | [Ontwikkelen met de doelgroep](https://www.tudelft.nl/inclusive-ehealth-guide/ontwikkelen/ontwikkelen-met-de-doelgroep) (Faber et al., 2023) |
|  | [Guidance on authorship with and acknowledgement of patient partners in patient-oriented research](https://link.springer.com/article/10.1186/s40900-020-00213-6) (Richards et al., 2020) |
| Ondersteun duurzame samenwerking | [Patient stakeholder engagement in research: A narrative review to describe foundational principles and best practice activities](https://onlinelibrary.wiley.com/doi/10.1111/hex.12873) (Harrison et al., 2019) |
|  | [Meaningful Inclusion of Partners in Decision Making](https://www.pcori.org/engagement-research/engagement-resources/foundational-expectations/meaningful-inclusion) (Patient-centered Outcomes Research Institute, 2025) |

**Stap 6: EVALUATIE**

*Evaluatie tijdens en na afloop van het project*

Hoe is het participatieproces (tot nu toe) verlopen?

| *Vragen ter overweging:*   - *Welke aspecten zijn belangrijk om te evalueren voor jou en voor de eindgebruikers?* - *Hoe verloopt het participatieproces?* - *Worden de vooraf gedefinieerde doelstellingen gehaald?* - *Komt het overeen met ieders verwachtingen en behoeften?* - *Zijn er verbeterpunten of elementen die behouden moeten blijven?* - *Wat is tot nu toe de toegevoegde waarde van participatie?* - *Hoe kunnen de resultaten of relaties uit deze samenwerking worden behouden?*   *Notities:* |
| --- |

| Aanbevelingen | Hulpmiddelen |
| --- | --- |
| Integreer voortdurende monitoring en evaluatie | [Patient stakeholder engagement in research: A narrative review to describe foundational principles and best practice activities](https://onlinelibrary.wiley.com/doi/10.1111/hex.12873) (Harrison et al., 2019) |
|  | [Ongoing Review & Assessment of Engagement](https://www.pcori.org/engagement-research/engagement-resources/foundational-expectations/ongoing-review-and-assessment) (Patient-centered Outcomes Research Institute, 2025) |
|  | [Patient resources](https://ossu.ca/for-patients/resources/) (Ontario SPOR SUPPORT Unit, n.d.) |
| Voer aan het einde van het project een evaluatie uit | [PPEET vragenlijst](https://doi.org/10.1007/s12508-021-00316-9) (Bavelaar et al., 2021) |
|  | [An Empirical‐Theoretical Analysis Framework for Public Participation in Environmental Impact Assessment](https://www.tandfonline.com/doi/abs/10.1080/713676582) (Palerm, 2000) |
|  | [A model and measure for quality service user involvement in health research](https://doi.org/10.1111/j.1470-6431.2010.00901.x) (Morrow et al., 2010) |
|  | [Patient stakeholder engagement in research: A narrative review to describe foundational principles and best practice activities](https://onlinelibrary.wiley.com/doi/10.1111/hex.12873) (Harrison et al., 2019) |
|  | [Patient resources](https://ossu.ca/for-patients/resources/) (Ontario SPOR SUPPORT Unit, n.d.) |
| Neem kennis en samenwerking mee naar vervolgprojecten | [Community-Based Participatory Research](https://doi.org/10.1037/amp0000167)  (e.g., Collins et al., 2018) |
|  | [Participatory action research](https://doi.org/10.1136/jech.2004.028662) (Baum et al., 2006) |

**References**

Agnello, D. M., Balaskas, G., Steiner, A., & Chastin, S. (2024). Methods Used in Co-Creation Within the Health CASCADE Co-Creation Database and Gray Literature: Systematic Methods Overview. *Interactive Journal of Medical Research*, *13*. <https://doi.org/10.2196/59772>

Bagley, H. J., Short, H., Harman, N. L., Hickey, H. R., Gamble, C. L., Woolfall, K., Young, B., & Williamson, P. R. (2016). A patient and public involvement (PPI) toolkit for meaningful and flexible involvement in clinical trials - a work in progress. *Res Involv Engagem*, *2*, 15. <https://doi.org/10.1186/s40900-016-0029-8>

Baum, F., MacDougall, C., & Smith, D. (2006). Participatory action research. *J Epidemiol Community Health*, *60*(10), 854–857. <https://doi.org/10.1136/jech.2004.028662>

Bavelaar, L., van Tol, L. S., Caljouw, M. A., & van der Steen, J. T. (2021). Nederlandse vertaling en eerste stappen in validatie van de PPEET om burger-en patiëntenparticipatie te evalueren. *TSG-Tijdschrift voor gezondheidswetenschappen*, *99*(4), 146–153.

Bonten, T. N., Rauwerdink, A., Wyatt, J. C., Kasteleyn, M. J., Witkamp, L., Riper, H., van Gemert-Pijnen, L. J., Cresswell, K., Sheikh, A., Schijven, M. P., Chavannes, N. H., & Group, E. H. E. R. (2020). Online Guide for Electronic Health Evaluation Approaches: Systematic Scoping Review and Concept Mapping Study. *J Med Internet Res*, *22*(8), e17774. <https://doi.org/10.2196/17774>

Brown, T., & Katz, B. (2011). Change by Design. *Journal of Product Innovation Management*, *28*(3). <https://doi.org/10.1111/j.1540-5885.2011.00806.x>

Citrienfonds. (n.d.). *eHealth methodology guide*. Retrieved August 2, 2025 from <https://citrienfonds-ehealth.nl/e-health-toolkit/onderzoek/e-health-evaluation-methodology/overview-of-methods/>

Collins, S. E., Clifasefi, S. L., Stanton, J., Straits, K. J. E., Gil-Kashiwabara, E., Espinosa, P. R., Nicasio, A. V., Andrasik, M. P., Hawes, S. M., Miller, K. A., Nelson, L. A., Orfaly, V. E., Duran, B. M., Wallerstein, N., & Board, L. A. (2018). Community-Based Participatory Research (CBPR): Towards Equitable Involvement of Community in Psychology Research. *American Psychologist*, *73*(7), 884–898. <https://doi.org/10.1037/amp0000167>

Dutch Oncology Research Platform. (2023). *Patiëntenparticipatie in Clinical Trial Roadmap*. Retrieved August 12, 2025 from <https://participatiekompas.nl/clinical-trial-roadmap>

Faber, J. S., Al-Dhahir, I., Kraal, J. J., Breeman, L. D., van den Berg-Emons, R. J. G., Reijnders, T., van Dijk, S., Janssen, V. R., Kraaijenhagen, R. A., Visch, V. T., Chavannes, N. H., & Evers, A. W. M. (2023). Guide Development for eHealth Interventions Targeting People With a Low Socioeconomic Position: Participatory Design Approach. *J Med Internet Res*, *25*, e48461. <https://doi.org/10.2196/48461>

Harrison, J. D., Auerbach, A. D., Anderson, W., Fagan, M., Carnie, M., Hanson, C., Banta, J., Symczak, G., Robinson, E., Schnipper, J., Wong, C., & Weiss, R. (2019). Patient stakeholder engagement in research: A narrative review to describe foundational principles and best practice activities. *Health Expect*, *22*(3), 307–316. <https://doi.org/10.1111/hex.12873>

ICM Opleidingen & Trainingen. (n.d.). *100 werkvormen voor docenten en trainers*. Retrieved August 12, 2025 from <https://www.icm.nl/extra/100-werkvormen-docenten-en-trainers/>

IDEO.org. (n.d.). *Define your audience*. Retrieved August 12, 2025 from <https://www.designkit.org/methods/define-your-audience.html>

INVOLV. (n.d.–a). *Kickstarter voor onderzoekers*. Retrieved June 2, 2025 from <https://www.involv.nl/advies-voor-onderzoekers/kickstarter>

INVOLV. (n.d.–b). *Rollen in participatie*. Retrieved August 12, 2025 from <https://www.involv.nl/trainingen/rollen-participatie-online-module>

INVOLV. (n.d.–c). *Trainingen*. Retrieved August 12, 2025 from <https://www.involv.nl/trainingen/trainingenoverzicht?page=4>

Kenniscentrum Revalidatiegeneeskunde Utrecht. (2019). *Participatiematrix*. Kenniscentrum Revalidatiegeneeskunde Utrecht. Retrieved 3 February from <https://www.kcrutrecht.nl/producten/participatiematrix/>

Kip, H., Beerlage-de Jong, N., van Gemert-Pijnen, L., & Kelders, S. M. (2025). The CeHRes Roadmap 2.0: Update of a Holistic Framework for Development, Implementation, and Evaluation of eHealth Technologies. *J Med Internet Res*, *27*(1), e59601. <https://doi.org/10.2196/59601>

Morrow, E., Ross, F., Grocott, P., & Bennett, J. (2010). A model and measure for quality service user involvement in health research. *International Journal Of Consumer Studies*, *34*(5). <https://doi.org/10.1111/j.1470-6431.2010.00901.x>

Ontario SPOR SUPPORT Unit. (n.d.). *Patient resources*. Retrieved August 12, 2025 from <https://ossu.ca/for-patients/resources/>

Palerm, J. R. (2000). An Empirical‐Theoretical Analysis Framework for Public Participation in Environmental Impact Assessment. *Journal of Environmental Planning and Management*, *43*(5). <https://doi.org/10.1080/713676582>

Patient-centered Outcomes Research Institute. (2025). *Engagement in Research*. Patient-centered Outcomes Research Institute. Retrieved 3 February from <https://www.pcori.org/engagement-research/value-engagement-research>

Patiëntenfederatie Nederland. (2025). *Vinden van patiëntenverenigingen*. Retrieved August 12, 2025 from <https://kennisbank.patientenfederatie.nl/app/answers/detail/a_id/29/~/vinden-van-pati%C3%ABntenvereniging>

Pharos. (n.d.). *eHealth of digitale zorg die iedereen kan gebruiken: hoe doe je dat?* Retrieved August 12, 2025 from <https://www.pharos.nl/ehealth-en-digitale-zorg-voor-iedereen/>

RE-AIM. (2025). *Interactive RE-AIM Planning Tool*. Retrieved August 4, 2025 from <https://re-aim.org/applying-the-re-aim-framework/re-aim-guidance/use-when-planning-a-project/planning-tool/>

Richards, D. P., Birnie, K. A., Eubanks, K., Lane, T., Linkiewich, D., Singer, L., Stinson, J. N., & Begley, K. N. (2020). Guidance on authorship with and acknowledgement of patient partners in patient-oriented research. *Res Involv Engagem*, *6*, 38. <https://doi.org/10.1186/s40900-020-00213-6>

Schoemaker, C. G., Richards, D. P., & de Wit, M. (2023). Matching researchers' needs and patients' contributions: practical tips for meaningful patient engagement from the field of rheumatology. *Annals of the Rheumatic Diseases*, *82*(3), 312–315. <https://doi.org/10.1136/ard-2022-223561>

Stappers, P., & Giaccardi, E. (2017). *Research through Design*. Soegaard, M. & Friis-Dam, R. Retrieved August 12, 2025 from <https://www.interaction-design.org/literature/book/the-encyclopedia-of-human-computer-interaction-2nd-ed/research-through-design>

Torfs, E., & Wismans, G. (2015). *Activerende werkvormen*. Retrieved August 12, 2025 from <https://teaching-and-learning-collection.sites.uu.nl/knowledge_item/activerende-werkvormen/>
